# Sustained, neuron-specific IKK/NF-κB activation generates a selective neuroinflammatory response promoting local neurodegeneration with aging

**DOI:** 10.1186/1750-1326-8-40

**Published:** 2013-10-12

**Authors:** Ayesha Maqbool, Michael Lattke, Thomas Wirth, Bernd Baumann

**Affiliations:** 1 Institute of Physiological Chemistry, Ulm University, Albert-Einstein-Allee 11, Ulm 89081 Germany

**Keywords:** Neuroinflammation, Neurodegeneration, NF-κB, IKK2, Bdnf, Spatial learning and memory, Dentate gyrus

## Abstract

**Background:**

Increasing evidence indicates that neuroinflammation is a critical factor contributing to the progression of various neurodegenerative diseases. The IKK/NF-κB signalling system is a central regulator of inflammation, but it also affects neuronal survival and differentiation. A complex interplay between different CNS resident cells and infiltrating immune cells, which produce and respond to various inflammatory mediators, determines whether neuroinflammation is beneficial or detrimental. The IKK/NF-κB system is involved in both production of and responses to these mediators, although the precise contribution depends on the cell type as well as the cellular context, and is only partially understood. Here we investigated the specific contribution of neuronal IKK/NF-κB signalling on the regulation of neuroinflammatory processes and its consequences. To address this issue, we established and analysed a conditional gain-of-function mouse model that expresses a constitutively active allele of IKK2 in principal forebrain neurons (IKK2^nCA^). Proinflammatory gene and growth factor expression, histopathology, microgliosis, astrogliosis, immune cell infiltration and spatial learning were assessed at different timepoints after persistent canonical IKK2/NF-κB activation.

**Results:**

In contrast to other cell types and organ systems, chronic IKK2/NF-κB signalling in forebrain neurons of adult IKK2^nCA^ animals did not cause a full-blown inflammatory response including infiltration of immune cells. Instead, we found a selective inflammatory response in the dentate gyrus characterized by astrogliosis, microgliosis and Tnf-α upregulation. Furthermore, downregulation of the neurotrophic factor Bdnf correlated with a selective and progressive atrophy of the dentate gyrus and a decline in hippocampus-dependent spatial learning. Neuronal degeneration was associated with increased Fluoro-jade staining, but lacked activation of apoptosis. Remarkably, neuronal loss could be partially reversed when chronic IKK2/NF-κB signalling was turned off and Bdnf expression was restored.

**Conclusion:**

Our results demonstrate that persistent IKK2/NF-κB signalling in forebrain neurons does not induce overall neuroinflammation, but elicits a selective inflammatory response in the dentate gyrus accompanied by decreased neuronal survival and impaired learning and memory. Our findings further suggest that chronic activation of neuronal IKK2/NF-κB signalling, possibly as a consequence of neuroinflammatory conditions, is able to induce apoptosis-independent neurodegeneration via paracrine suppression of Bdnf synthesis.

## Background

Neuroinflammation is a common hallmark of several CNS disorders, which is characterized by the upregulation of proinflammatory cytokines and chemokines such as *Tnf, Ccl2* and *Cxcl10* as well as the infiltration of activated immune cells [1].

The activation of the NF-κB family of transcription factors is a key step in the regulation of inflammatory and immune responses. However, these proteins also regulate gene expression in a variety of other physiological processes like cell proliferation, differentiation and survival, as well as specific CNS functions including learning and memory [2]. In resting cells, NF-κB dimers are sequestered in the cytosol by inhibitory proteins of the IκB family. The crucial step in NF-κB activation is the phosphorylation of IκB proteins by the activating IκB kinase complex. IKK2 is the critical kinase subunit inducing the canonical signalling pathway, which is essentially involved in the regulation of inflammation. Phosphorylation of inhibitory IκB proteins initiates their ubiquitination and subsequent proteosomal degradation, followed by the release and nuclear translocation of active NF-κB dimers, which then induce the expression of NF-κB target genes [3-5].

Members of the IKK/NF-κB system are widely expressed in the nervous system and different factors stimulate NF-κB activation in the CNS, including damage-associated molecular patterns, pathogen-associated molecular patterns, cytokines, chemokines, neurotransmitters, neurotrophic factors and neurotoxins. NF-κB is activated both under physiological conditions, e.g. by synaptic activity, as well as in pathological conditions [6-8]. Previous studies reported that the IKK/NF-κB signalling system is deregulated in various neuroinflammatory conditions as Alzheimer’s disease (AD), Huntington’s disease (HD), stroke, hydrocephalus and schizophrenia [9-15]. Depending on the cell type and pathophysiological context, both protective and deleterious roles of NF-κB signalling were found in CNS diseases, e.g. in ischemic injury [16,17].

We have previously shown that suppression of IKK/NF-κB signalling in neurons reduces infarct formation in an animal model of stroke. Vice versa ectopic activation of IKK2 in a similar context increases the infarct size after cerebral ischemia [9]. These findings indicate a central role of neuronal NF-κB in the regulation of cell survival in acute stroke pathogenesis.

In order to characterize the detailed role of neuronal NF-κB in the pathogenesis of chronic neurodegenerative disorders, we analysed the consequences of persistent NF-κB activation in neurons using the IKK2^nCA^ model. As NF-κB activation is sufficient to induce strong inflammatory processes in various cell types and tissues [11,18-20], we particularly asked whether chronic NF-κB activation in neurons is sufficient to drive a neuroinflammatory response on its own and if so, what are the pathological consequences.

Unexpectedly, IKK2^nCA^ animals did not show massive signs of neuroinflammation such as prominent proinflammatory cytokine expression and infiltration of immune cells. Interestingly, they exhibited downregulation of the neurotrophic factor Bdnf, which correlates with an impairment of cognitive functions and degeneration of the dentate gyrus.

## Results

### Conditional expression of IKK2-CA in principal forebrain neurons

**Figure 1:**
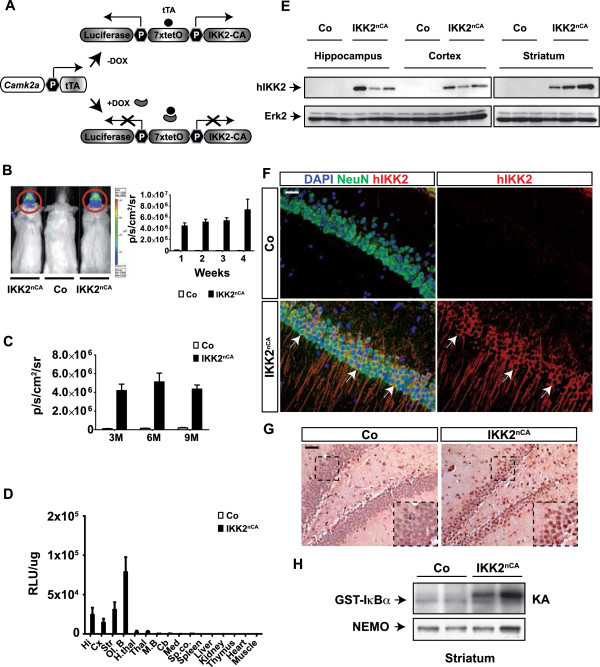
**Gain-of-function model for conditional IKK/NF-κB signalling in excitatory forebrain neurons. (A)** Generation of the conditional transgenic IKK2^nCA^ mouse model using the tetracycline-regulated gene expression system (tet-off). The *Camk2a* promoter drives the expression of the tetracycline transactivator protein (tTA). The tTA-activated promoter (7xtetO) directs the transcription of luciferase and the constitutively active IKK2-CA allele (IKK2^nCA^). Doxycycline (DOX) administration blocks transgene expression. **(B)** In vivo luciferase measurement of IKK2^nCA^ mice indicates forebrain-restricted transgene expression (left panel). Luciferase expression (p/s/cm2/sr) showed rapid transgene expression already within one week after DOX removal that sustains with age (right panel) (n = 14). **(C)** Robust transgene expression over time. 3M, 6M, 9M = 3, 6 and 9 month (n = 13). **(D)** Luciferase activity was measured in different organs and brain regions of 3M old mice. RLU/μg = relative light units per μg protein (n = 4). **(E)** IKK2-CA transgene expression (hIKK2) was monitored in different brain regions of 3M old mice. Erk2 is used as loading control (n = 3). **(F)** Immunohistochemical analysis of IKK2-CA expression (hIKK2, red; NeuN; green; 9M). Arrows indicate IKK2-CA expression in CA1 co-expressed with NeuN (yellow). DAPI (blue). Scale bar: 50 μm. **(G)** IKK2^nCA^ mice (9M) show increased nuclear RelA staining in the DG. Inserts depict magnification of marked areas. **(H)** Increased IKK activity in striatal lysates of IKK2^nCA^ mice (3M) was monitored by in vitro kinase assay using GST-IκBα as IKK substrate. Immunoprecipitated NEMO protein levels serve as loading control. **(A**-**H)** Co = wild type and single transgenic littermates, IKK2^nCA^ = mice with neuron-specific IKK2-CA expression. Hi, hippocampus; cx, cortex, str, striatum; Ol.B, Olfactory bulbi; H.thal, hypothalamus; Thal, thalamus, M.B, midbrain; Cb, cerebellum; Med, medulla; Sp.co., spinal cord. All data are shown as mean ± SEM. All *p*-values are derived from two-tailed-unpaired student’s *t* test.

To study the consequences of chronic NF-κB activation in forebrain neurons, we used the previously generated IKK2^nCA^ mouse model [9]. This conditional gain-of-function model co-expresses a constitutively active allele of IKK2 (IKK2-CA) and a luciferase reporter gene under the control of the *Camk2a* promoter in a tetracycline-regulated manner (CamK2a-tTA x luciferase-(tetO)_7_-IKK2-CA, called IKK2^nCA^) (Figure 1A). To avoid any influences of transgene expression on brain development, animals were bred and housed in the presence of doxycycline (DOX) up to the age of 4 weeks.

To determine the transgene expression kinetics after DOX withdrawal in IKK2^nCA^ mice, we first analysed the activity of the co-expressed reporter gene luciferase using *in vivo* bioluminescence measurement (IVIS). We found a CNS-restricted luciferase activity, beginning within the first week of DOX withdrawal, which was then stable for at least 8 months (Figure 1B and C), indicating a rapid and robust transgene expression. To further analyse the transgene expression pattern and its detailed spatial resolution, we measured luciferase activity in protein lysates from various brain regions and control organs. As expected from the forebrain specific expression pattern of *Camk2a*[21], luciferase activity depicted transgene expression in the hippocampus, cortex, striatum and olfactory bulb, but no or only a minor expression in other brain regions and peripheral organs (Figure 1D). Transgene expression was confirmed in the hippocampus, striatum, and cortex by protein immunoblot analysis (Figure 1E).

We next investigated cell type specificity of transgene expression by immunofluorescence co-staining with the neuronal marker NeuN. As shown for the hippocampal CA1 region, expression of IKK2^nCA^ was restricted to NeuN positive neurons (Figure 1F). To assess the functional consequences of IKK2-CA expression, subcellular localisation of the NF-κB subunit p65/RelA was analysed. RelA staining revealed a strong nuclear reactivity in neurons of the DG (Figure 1G) and of the CA1 region, cortex and striatum of IKK2^nCA^ mice (see Additional file 1) indicating neuronal NF-κB activation in these animals. Moreover, IKK activity was elevated in striatal lysates of IKK2^nCA^ mice as measured by the phosphorylation level of the IKK substrate GST-IκBα (Figure 1H).

### IKK2^nCA^ mice do not show prominent signs of neuroinflammation

**Figure 2:**
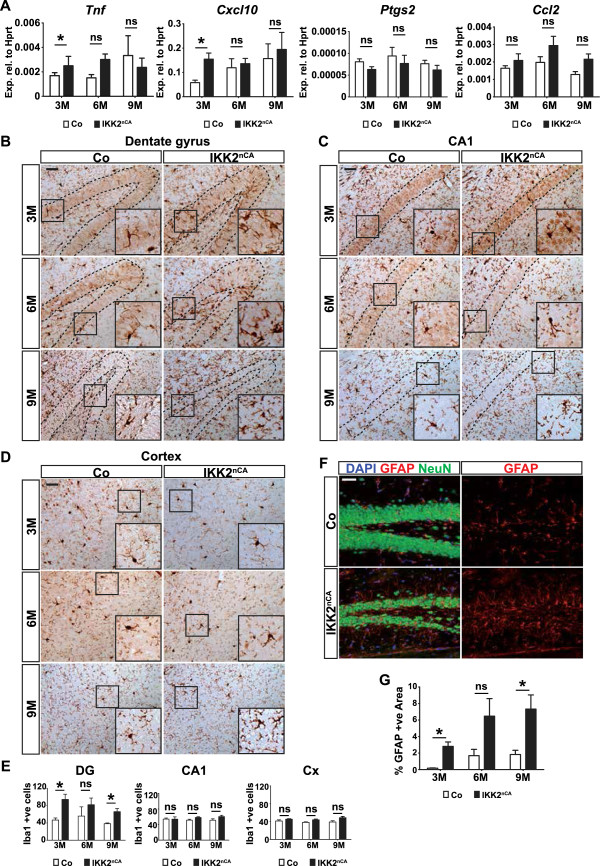
**IKK2**^**nCA **^**mice show selective neuroinflammation. (A)** mRNA was isolated from hippocampus of IKK2^nCA^ mice and qRT-PCR analysis for various cytokines dipicts mild upregulation of *Tnf* and *Cxcl10* only at 3M, whereas there were no alterations for *Ptgs2* and *Ccl2*. Data presented are calculated relative to *Hprt* expression. **(B)** IKK2-CA induces microgliosis in the DG of IKK2^nCA^ mice at 3M and 9M of age as represented by a larger population of Iba-1-positive microglia. **(C)** Representative images of Iba-1 immunohistochemistry show comparable number of microglia in the CA1-region of control and IKK2^nCA^ mice at different ages. **(D)** Representative images of Iba-1 immunocytochemistry in brain sections indicate a normal microglial morphology and density in cortex of IKK2^nCA^ mice at different ages. **(E)** Quantification of Iba1 positive cells in control and IKK2^nCA^ mice at the age of 3, 6 and 9 months from B, C and D shows moderate microgliosis only in the DG, but not in the CA1-region and cortex (n = 3-4). **(F)** IKK2^nCA^ mice exhibit astrogliosis as shown by the increased reactivity in the DG by immunofluorescent staining for GFAP, the astroglial marker (Age = 9M). GFAP positive area was measured using ImageJ64 software (n = 3). **(G)** Quantification of the GFAP positive area in the DG represents significantly enhanced GFAP immunoreactivity at 3M and 9M, as well as a marked tendency of an increased GFAP positive area at 6M, indicating astrogliosis (n = 3-4). Co = wild type and single transgenic littermate controls, IKK2^nCA^ = transgenic mice with neuron-specific expression of constitutively active IKK2. **(B**-**D)** Inserts at the right bottom depict the magnification of the marked areas. All data are shown as mean ± SEM. All *p*-values are derived from two-tailed-unpaired student’s *t* test. * *p* < 0.05, ** *p* < 0.01. Scale bar: 50 μm.

NF-κB is the key regulator of inflammation [3], we therefore analysed the expression of proinflammatory factors, in particular *Tnf, Ccl2*, *Ptgs2* and *Cxcl10*, which are well-characterized target genes of NF-κB [22]. In contrast to other models [11,18-20,23], and with the exception of a mild upregulation of *Tnf *and *Cxcl10* in the hippocampus of 3 months old IKK2^nCA^ mice, no upregulation of *Ccl2* and *Ptgs2* could be detected (Figure 2A). We also did not observe any elevation of other proinflammatory factors like *Ccl5* and *Il6* (see Additional file 2A). To further characterize inflammatory gene expression in the CNS of IKK2^nCA^ mice Tnf-α, co-immunostaining with NeuN was conducted in different brain subregions. Interestingly, Tnf-α immunoreactivity is especially increased within hilar neurons of the DG, but not in the CA1-region or in the cortex of 9 months old IKK2^nCA^ mice (see Additional file 2B). To confirm the subregion-specific expression of *Tnf* RNA from the DG and Cornu Ammonis (CA) region was isolated. Precise subregional RNA isolation was certified by qRT-PCR analysis for the DG-specific gene, Tryptophan 2,3-dioxygenase (*Tdo2*) and the CA1-specific gene, Meis1-related protein 1b (*Mrg1b*) (Additional file 2C) [24]. We observed an increase in *Tnf* levels exclusively in the DG, which is lacking in the CA-region. *Cxcl10* expression shows a tendency to be upregulated in both sub-regions, whereas *Ccl2* and *Il6* are not altered at the age of 9 months (Additional file 2C).

Since neuroinflammation involves the activation of microglia, we assessed their status in different forebrain regions by Iba1 immunostaining. Consistent with the results seen in Figure 2A, Iba1 staining only revealed selective microgliosis in the dentate gyrus (DG), whereas other IKK2-CA-expressing brain regions like cortex and the hippocampal CA1-region lacked microglial activation at any timepoint analysed (Figure 2B-E). Astrocytes also get activated under inflammatory conditions, a process that is characterized by hypertrophic astroglia and an upregulation of the astrocyte-specific intermediate filament protein GFAP. Similar to the previous findings, astrogliosis was detected only in the DG of IKK2^nCA^ mice (Figure 2F, G). We could not detect infiltration of CD45^+^ immune cells and upregulation of *Lcn2*, an inflammatory marker gene (Additional file 3) found in a different neuroinflammatory mouse model with immune cell infiltration [11]. Therefore we conclude that IKK2-mediated NF-κB activation in excitatory forebrain neurons is not sufficient to induce a full-blown inflammatory response.

### Persistent IKK/NF-κB signalling interferes with hippocampus dependent spatial learning

**Figure 3:**
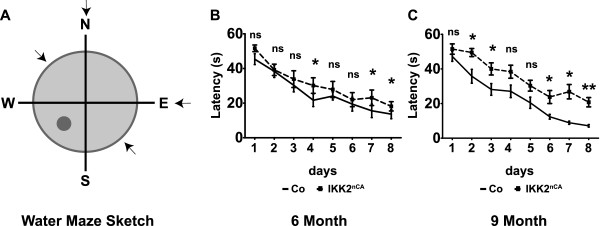
**Forebrain-specific IKK2-CA expression impairs hippocampus-dependent spatial learning. (A)** Scheme of a circular pond used for the Morris water maze paradigm. N, S, E and W mark the orientation of the pond. N (north), S (south), E (east) and W (west). The platform was localised in the centre of the SW quadrant for eight consecutive training days. Arrows mark the different starting positions used in the experiments. **(B)** Deficit in hippocampus dependent spatial learning of IKK2^nCA^ mice is deduced from requirement of more time to find the hidden platform in MWM task as compared to the controls at 6M age (n = 14-16). **(C)** Impaired learning performance of 9M old IKK2^nCA^ mice indicated by their longer escape latency than the littermates during 8 days’ testing time (n = 13). **(B**-**C)** All mice were adult males. All *p*-values are derived from two-tailed-unpaired student’s *t* test, Co = wild type and single transgenic littermate controls, IKK2^nCA^ = transgenic mice with neuron-specific expression of constitutively active IKK2. All data are shown as mean ± SEM. ** *p* < 0.05, ** *p* < 0.01, *** *p* < 0.001.

Several studies demonstrated that the IKK/NF-κB system is involved in the regulation of neuronal differentiation including neurite outgrowth, synaptic plasticity and synapse formation [6-8,25,26]. As interference with IKK/NF-κB signalling was found to impair learning and memory in various experimental paradigms [26-33], we asked whether there is an improvement of learning behaviour in our gain-of-function model. For this purpose, IKK2^nCA^ mice were exposed to the Morris water maze task (MWM), which is a test for hippocampus-dependent spatial learning [34]. Here, the animals were trained to find a hidden platform in a water-filled pool within 60s (Figure 3A). Both, control and IKK2^nCA^ mice learned to find this platform but IKK2^nCA^ mice required longer time to navigate and to reach the platform as compared to control littermates (Figure 3B, C). This implied that persistent neuronal IKK2/NF-κB activation does not enhance, but rather interferes with spatial cognitive abilities in the MWM tasks.

### Chronic IKK/NF-κB signalling reduces Bdnf expression in the forebrain of IKK2^nCA^ mice

**Figure 4:**
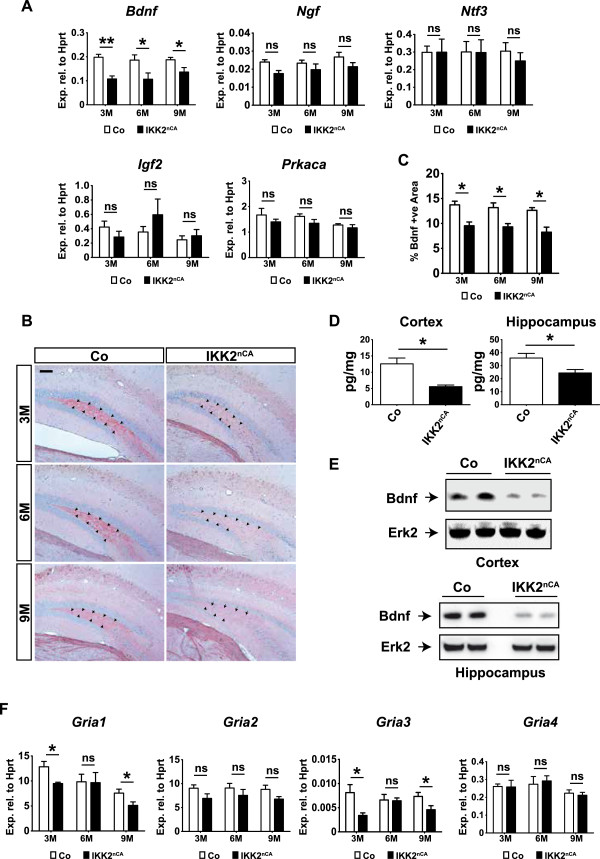
**Bdnf expression is decreased in the hippocampus and cortex of IKK2**^**nCA **^**mice. (A)** qRT-PCR analysis indicates significantly reduced levels of *Bdnf* mRNA in the hippocampus but no changes for the other analysed memory-associated genes as *Ngf*, *Ntf3*, *Igf2* and *Prkaca* were detected (n = 4-7). *P*-values are derived from two-tailed-unpaired student’s *t* test. **(B)** Representative images from immunostaining of IKK2^nCA^ mice brains reveal a decline in Bdnf immunoreactivity in the hilus of the dentate gyrus. Inward arrows point towards the expression of Bdnf in the hilus (n = 3-5). Scale bar: 100 μm. **(C)** Quantification of Bdnf immunoreactivity by ImageJ64 represents decreased Bdnf expression in the dentate hilus of IKK2^nCA^ mice at 3M, 6M and 9M age. *p*-values are derived from one way ANOVA Bonferroni’s Multiple Comparison test. **(D)** Measurement of Bdnf concentration by ELISA indicates significant decrease in total Bdnf levels in the cortex and hippocampus of IKK2^nCA^ mice at 6M of age (n = 4). *P*-values are derived from two-tailed-unpaired student’s *t* test. **(E)** Western blot analysis demonstrates Bdnf reduction in cortex and hippocampus of IKK2^nCA^ mice at the age of 9M. Erk2 is used as loading control. **(F)** qRT-PCR shows that decreased Bdnf levels are associated with a reduced expression of *Gria1* (GluR1) and *Gria3* (GluR3) type AMPA receptors in the hippocampus at 3 and 9 months of age but no alterations are detected for *Gria2* (GluR2) and *Gria4* (GluR4) in all three age groups. *p*-values are derived from two-tailed-unpaired student’s *t* test. **(A**-**F)** Co = wild type and single transgenic littermate controls, IKK2^nCA^ = transgenic mice with neuron-specific expression of constitutively active IKK2. All data are shown as mean ± SEM. * *p* < 0.05, ** *p* < 0.01.

Since IKK2^nCA^ mice exhibit deficits in spatial learning, we addressed putative mechanisms possibly affecting neuronal function, synaptic plasticity and cognition in these animals. Therefore, we investigated the expression of genes critically involved in the regulation of synaptic plasticity, neuronal differentiation and survival [29,35-37]. Interestingly, we detected a reduced expression of the neurotrophic factor *Bdnf* in hippocampal samples by qPCR, whereas other neurotrophins (*Ngf* and *Ntf3*), and memory-associated genes like *Igf2* and *Prkaca* were not deregulated (Figure 4A). To confirm this finding, we also measured Bdnf levels by immunohistochemistry, immunoblotting and ELISA. Indeed, a decreased Bdnf reactivity could be observed in the hilus of the DG of IKK2^nCA^ mice already at the age of 3 months and also in older animals (Figure 4B, C). DG-specific reduction of Bdnf expression was confirmed by subregion-specific mRNA expression analysis (Additional file 4). Moreover, ELISA indicated a reduced level of Bdnf in cortex and hippocampus at the age of 6 months (Figure 4D), which was confirmed by immunoblotting in hippocampal and cortical lysates at the age of 9 months (Figure 4E). One effector function of Bdnf is the regulation of α-amino-3-hydroxy-5-methyl-4-isoxazolepropionic acid receptor (AMPAR) expression [38,39] and AMPAR subunits are known to be expressed in the hippocampus [40]. Interestingly, we observed a mild decrease in *Gria1* and *Gria3* expression at 3 and 9 months of age, whereas no obvious changes in *Gria2* and *Gria4* levels were noticed (Figure 4F). Analysis of mRNA from the DG and CA-region showed DG-specific downregulation of *Gria3* AMPA receptors in the IKK2^nCA^ mice as compared to the littermates. *Gria1* reduction was also observed in the CA-region, whereas *Prkaca* levels remain unaltered (Additional file 4).

### IKK2^nCA^ mice develop a granular cell layer specific degeneration of the dentate gyrus

We then asked whether the decreased Bdnf levels have any effect on neuronal survival and analysed the hippocampi of age-matched control and IKK2^nCA^ mice by cresyl violet staining.

**Figure 5:**
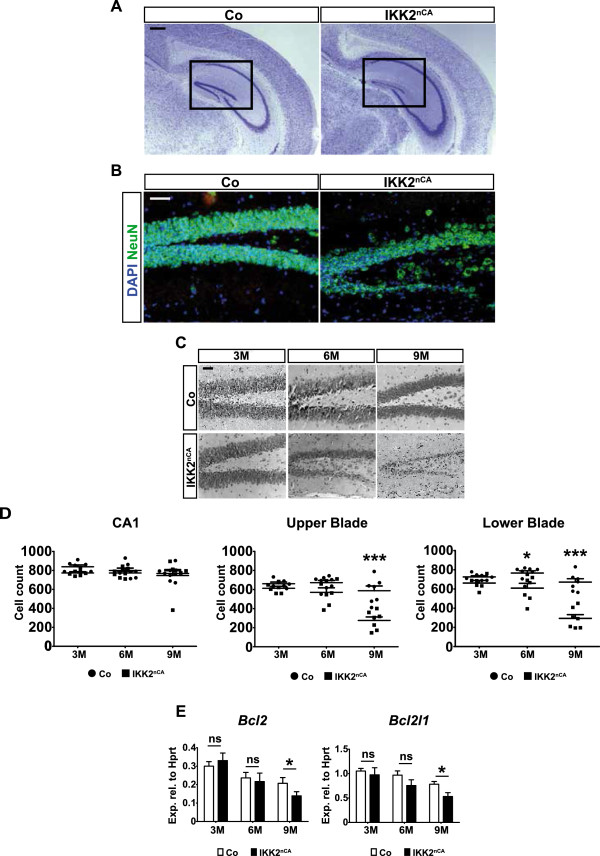
**IKK2-CA expression results in a degeneration of DG neurons in the GCL. (A)** Nissl staining illustrates degeneration in the DG of IKK2^nCA^ mice at the age of 9M (n = 7). **(B)** Immunofluorescent staining of kryosections with NeuN (green) confirms that the cells lost in the dentate gyrus are GCL neurons. Co-staining with DAPI (blue) was performed for visualising the nuclei (n = 3). **(C)** Representative images of Nissl-stained sections depict a progressive, age-dependent degeneration in the DG of IKK2^nCA^ mice from 3M to 9M of age (n = 6-8). **(D)** Quantification of cell numbers in the CA1 region and DG manifest an ongoing cell loss specifically in both blades of the dentate gyrus in a progressive manner (n = 6-8). **(E)** The anti-apoptotic genes *Bcl2* and *Bcl2l1* (*Bcl-xL*) are downregulated in the hippocampus of IKK2^nCA^ mice at 9M as indicated by qRT-PCR analysis (n = 4-7). **(A**-**E)** Co = wild type and single transgenic littermate controls, IKK2^nCA^ = transgenic mice with neuron-specific expression of constitutively active IKK2. Scale bar: 50 μm. All data are shown as mean ± SEM. *P*-values are derived from two-tailed-unpaired student’s *t* test. * *p* < 0.01.

Notably, we found a pronounced atrophy in both blades of the DG of IKK2^nCA^ at the age of 9 months (Figure 5A). Immunostaining with the neuronal marker NeuN indicated that atrophy depends on the loss of neurons located in the granular cell layer (GCL) of the dentate gyrus (Figure 5B), which is involved in regulation of learning and memory in a Bdnf dependent manner [41]. The quantification of the neurons in the DG revealed a progressive cell loss from 3 months to 9 months of age (Figure 5C and D). The decrease in cell number gets significant at 6 months when 20% of the cells are lost in the lower blade, which at 9 months culminates to a loss of 53% cells in the upper and 56% in lower blade. Remarkably, analysis of apoptosis by cleaved caspase-3 immunofluorescent staining or TUNEL assay did not reveal gross alterations between control and IKK2^nCA^ mice (Additional file 5). However, further analysis revealed an increased number of Fluoro-jade-positive neurons and neurites specifically in the DG of IKK2^nCA^ mice but not in the CA1, cortex and olfactory bulb (Additional file 6A-E). These findings indicate that most of the deleterious outcome associated with cell loss is due to apoptosis-independent neuronal degeneration and is restricted to the DG. Consistent with the lack of Fluoro-jade staining, histological analysis did not show significant changes in the thickness of primary cortex and granular cell layer (GCL) of the olfactory bulbs (Additional file 7A-D). The olfactory bulbs were also devoid of any changes in Bdnf and RNA expression (Additional file 6F).

Bdnf is able to promote neuronal survival via the expression of Bcl-2 [42]. In line with the pronounced loss of neurons in the DG, we detected a downregulation of *Bcl2* and another important pro-survival gene, *Bcl2l1* (Bcl-xL) at 9 months of age (Figure 5E). A subregion-specific qRT-PCR analysis demonstrated a reduction of *Bcl2l1* both in the DG and CA of IKK2^nCA^ mice whereas *Bcl2* expression was only tendentially decreased in this analysis (Additional file 7E).

### Replenishment of DG neurons by blocking IKK2-CA expression

**Figure 6:**
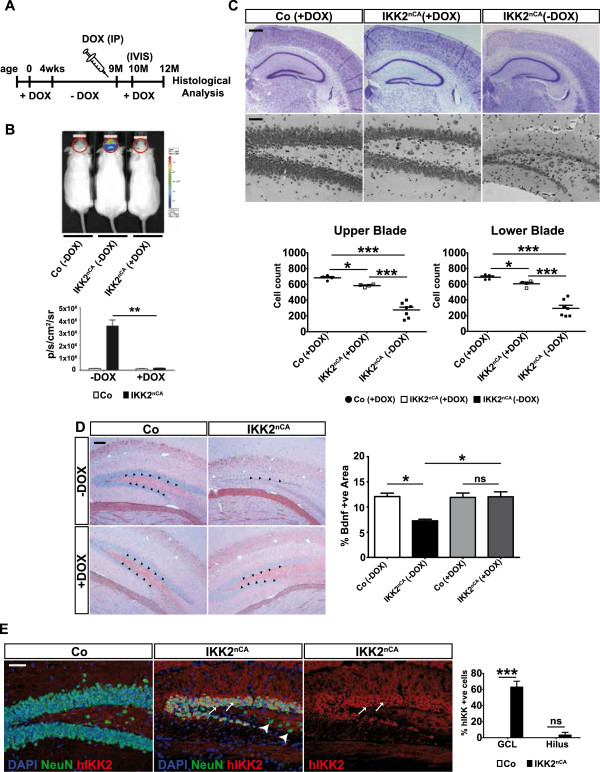
**DOX administration turns off transgene expression and restores the structure of the dentate gyrus in IKK2**^**nCA **^**animals. (A)** Experimental design indicating schedule of DOX administration to mice for transgene inactivation. **(B)** Representative image of Co and IKK2^nCA^ animals (treated as indicated) obtained by IVIS (upper panel). Quantification of in vivo luciferase activity demonstrates inactivation of transgene expression in IKK2^nCA^ mice by DOX application (lower panel; n = 4). All p-values are derived from two-tailed-unpaired student’s t test. **(C)** Cresyl violet stained sections show restoration of the DG morphology in IKK2^nCA^ mice after administrating DOX for 3 months. Quantification of cell number reveals that the number of neurons in GCL of IKK2^nCA^ mice recovers upon DOX treatment. All p-values are derived from two-tailed-unpaired student’s t test (n = 4). **(D)** Representative images from DG of IKK2^nCA^ mice indicate reversal of Bdnf expression after transgene inactivation. Arrows are pointing towards the hilus of the DG indicating Bdnf expression. Quantification of Bdnf immunoreactivity indicates reversal of Bdnf expression in the dentate hilus of IKK2^nCA^ mice after DOX treatment. *P*-values are derived one-way ANOVA Bonferroni’s Multiple Comparison test (n = 4). **(E)** Co-immunostaining of brain sections with hIKK2 transgene (red), and NeuN (green) demonstrating that only GCL neurons express hIKK2 (white arrows) whereas NeuN positive neurons in the hilus of DG (arrow heads) do not show any transgene expression (co-expression is shown in yellow). All fluorescent images show DAPI (blue) costaining. **(A**-**E)** Co = wild type and single transgenic littermates, IKK2^nCA^ = transgenic mice with neuron-specific expression of constitutively active IKK2. Scale bars: B, E: 50 μm, C: 100 μm. All data are shown as mean ± SEM. * *p* < 0.05, ** *p* < 0.01.

Persistent neuronal IKK/NF-κB signalling resulted in an age-dependent decline in cell number of the DG that is accompanied by microgliosis, astrogliosis and reduced Bdnf levels. To test whether this cell loss can be stopped or reversed, we blocked transgene expression in IKK2^nCA^ mice at an age with prominent DG degeneration by DOX application (Figure 6A). Downregulation of transgene expression was monitored by IVIS and a complete absence of the reporter gene luciferase was ascertained after 4 weeks of DOX administration in IKK2^nCA^ mice (Figure 6B). Cresyl violet staining and cell counting demonstrated a replenishment of neurons in the DG to 85% in the upper and to 87% in the lower blade after 3 months of continuous DOX treatment of IKK2^nCA^ mice when compared to the cell number of DOX treated control animals (Figure 6C). Intriguingly, Bdnf levels also recovered in the hilus region upon DOX application as compared to control and untreated age-matched animals (Figure 6D). When we monitored IKK2-CA transgene expression *in situ* in this brain region, we identified only a limited amount of IKK2-CA-positive cells in the hilus. This finding suggests that the reduced Bdnf expression in the hilus is not regulated directly by IKK2-CA but is rather controlled in a paracrine fashion (Figure 6E).

To investigate potential mechanisms underlying the structural reconstitution of the DG, we performed Ki67 staining, which indicated an increase in Ki67-immunoreactive cells in the DG of DOX treated IKK2^nCA^ mice (Additional file 8). This implies that IKK2-CA transgene inactivation and subsequent regain of Bdnf expression enhances adult neurogenesis in the GCL of the DG.

## Discussion

The IKK/NF-κB signalling system is proposed to be critically involved in the pathogenesis of various neurological diseases [8]. On the one hand, it is well characterized as a central regulator of inflammatory responses by controlling the expression of multiple proinflammatory acting genes [3,22]. On the other hand, IKK/NF-κB signalling is crucially involved in neuronal differentiation and various CNS functions [6-8]. However, due to its complex regulation in different cell types and diverse responses to different physiological and pathological conditions, the precise function of the IKK/NF-κB system in CNS physiology and pathology is only partially understood.

Former studies suggested an ambivalent role of the IKK/NF-κB system in the pathogenesis of neurological disorders [8,43]. Due to its proinflammatory function, NF-κB activation is able to trigger neuronal dysfunction, aging and cell death, thereby increasing severity of CNS diseases [8,11,44,45]. In contrast, NF-κB activation can also mediate neuroprotection [6,8,46]. Previously, we found that IKK2/NF-κB activation in neurons increases tissue damage in a mouse model of stroke, probably by enhancing the overall neuroinflammatory process elicited by this acute insult [9]. Therefore, we wanted to further investigate the role of IKK2-mediated neuron-specific NF-κB activation in the induction of neuroinflammatory responses using the IKK2^nCA^ model. We hypothesized that constitutive IKK2 activation in neurons is sufficient to induce inflammation, as it was demonstrated in several non-neural cell types as well as in astrocytes [11,18-20,23]. However, with the exception of microgliosis and astrogliosis observed in the DG, neuron-specific IKK2 activation did not result in a prominent inflammatory phenotype including infiltration of immune cells. Consistently, typical proinflammatory NF-κB target genes like *Ccl2*, *Tnf*, *Ptgs2, Lcn2* and *Cxcl10* that are highly expressed in other inflammatory conditions are either moderately or not induced in the IKK2^nCA^ model. This argues for a specific function of IKK2/NF-κB signalling in neurons.

What could be the reason for this unexpected response? As NF-κB is activated by synaptic signalling, such kind of NF-κB activation in neurons would already create a proinflammatory environment under the physiological conditions of neurotransmission. Vice versa, inflammation-mediated NF-κB activation in neurons would lead to functional conflicts like deregulation of NF-κB-mediated neurite outgrowth and synaptic plasticity. Therefore, a functional separation of neuronal IKK/NF-κB signalling versus inflammatory IKK/NF-κB signalling in other cells could be of physiological advantage. Several studies showed important functions of NF-κB in neuronal differentiation, including neurite outgrowth, formation and remodelling of synaptic connections, axogenesis and neuronal function, e.g. hippocampal learning and memory formation [6,26,28,47-50]. These studies are mainly based on experimental approaches inhibiting the IKK/NF-κB signalling system in neurons, therefore it could be anticipated that neuronal IKK/NF-κB activation might result in a phenotype that improves neuronal survival and cognitive capabilities. However, this idea appears to be in stark contrast to our findings. One plausible explanation for this discrepancy could be the duration of IKK/NF-κB signalling. In our model we induce permanent IKK/NF-κB activation over weeks, whereas in the physiological context of learning and memory rather a transient or repetitive activation is known to occur which is e.g., elicited by the neurotransmitter glutamate, known to induce NF-κB in synaptic signalling [51,52]. As excessive glutamate signalling results in excitotoxic cell death [53], we can speculate that the constitutive NF-κB activation in our model is probably detrimental to the DG neurons. Interestingly, pharmacological inhibition of IKK2 was able to block NMDA-induced excitotoxic cell death in hippocampal neurons and oligodendrocytes [54]. In line with the view that especially a transient NF-κB activation kinetic improves neuronal differentiation, Russo et al. shows that stereotactic application of a virus expressing IKK2-CA to the nucleus accumbens leads to spine formation within a short time of 3 days [50].

The adequate function of the adult dentate gyrus depends on both healthy mature granule cells as well as ongoing neurogenesis [55] and NF-κB was shown to be critically involved in different aspects of adult neurogenesis using loss-of-function approaches [28,56]. Since we only see neurodegeneration in the DG, a hypothesis might be that constitutive IKK2 activity also interferes with neurogenesis, which then results in a depletion of neurons in the GCL. However, a roughly 50% reduction in the cell count at 9M is difficult to explain solely by blockade of ongoing adult neurogenesis in the IKK2^nCA^ model but rather suggests active neurodegeneration. Furthermore, we could observe increased evels of Ki67-positive cells upon transgene inactivation arguing for elevated neurogenesis that may account for an active regeneration process of the DG rather than simple prevention of further neurodegeneration.

Imielski et al. [28] showed that the structural degeneration of the DG depends on apoptotic cell death, which was not detected in our model as measured by cleaved caspase-3 and TUNEL assay (Additional file 5). Instead, we could identify degenerating neurons in the DG but not in other brain regions by Fluoro-jade staining suggesting that IKK2-CA induces cell death but this cell death is independent of apoptosis or is due to a very slow rate of apoptosis that may escape detection. So far it remains largely open why this degeneration process is specific to the DG. However, our findings implicate that a combination of Bdnf decrease and Tnf increase (and possibly changes in other so far unkown factors) may account for the selective neurodegeneration of the dentate gyrus in the IKK2^nCA^ model. The structural restoration of the dentate gyrus after transgene inactivation in both models implies that fine balanced levels of NF-κB are required for appropriate neuronal survival and homeostasis in this brain region. Therefore, reactivation of the IKK/NF-κB system for therapeutic measures of neuro-regeneration in the context of dementia-associated diseases as suggested by Imielski et al. [28] is apparently critical and surely dose-dependent.

The neurodegenerative effect of constitutive IKK2 signalling could be due to the composition of the activated NF-κB dimers. In IKK2^nCA^ mice the canonical NF-κB pathway is active, most likely leading to the nuclear translocation of p65 containing dimers that are found to regulate apoptosis associated genes [57]. Also, there might be an under-representation of c-Rel containing dimers, which are known to promote neuronal survival by enhancing *Bcl2l1* transcription [57]. Corresponding to this, a downregulation of pro-survival genes like *Bcl2* and *Bcl2l1* was detected at older age in IKK2^nCA^ mice, although both of these are regulated by NF-κB [58,59]. Moreover, the decreased Bdnf expression in IKK2^nCA^ mice can be proposed as a potential mechanism that interferes with neuronal survival [60] because it also correlates with a decline in *Bcl2* and *Bcl2l1* levels [42]. Bdnf is well known to regulate cognitive tasks, synaptic plasticity and neuronal survival by activating its receptor TrkB [39,41,60,61] and its expression is compromised in brain disorders as AD, HD, Rett syndrome and schizophrenia [62,63]. Thus, the reduced levels of Bdnf and Bdnf-regulated AMPA receptors might attribute to the impaired hippocampal learning and the atrophy of the DG observed in our model.

Nevertheless, other factors may also contribute to the impaired learning and atrophy of the dentate gyrus. There is the possibility that the microgliosis and astrogliosis observed in the DG are sufficient to cause the neurodegeneration in IKK2^nCA^ mice [64,65]. Together with the elevated Tnf levels, such kind of inflammatory processes may influence learning and memory as well as neuronal survival. This might contribute to the observed phenotype, although the importance of low-grade neuroinflammation for learning and memory and neurodegeneration is still controversially discussed [66].

What is the underlying molecular mechanism resulting in reduced Bdnf, *Bcl2* and *Bcl2l1* expression in IKK2^nCA^ mice? The observed downregulation for Bdnf is rather surprising, as Bdnf is an NF-κB target gene in astrocytes [67] and would therefore expected to be rather upregulated in neurons, too. Although we did not investigate the mechanism behind this repression of Bdnf in IKK2^nCA^ mice, previous studies identified a similar kind of downregulation of target genes by NF-κB, e.g. in the case of hypoxia, *Tnf*-dependent EAAT2 expression, or in the regulation of anti-apoptotic genes after treatment of cells with DNA-damaging agents [68-70]. Campbell et al. showed that the cytotoxic stimuli like ultraviolet light (UV-C), and daunorubicin, downregulated the expression of anti-apoptotic NF-κB target genes like *Bcl2, Bcl2l1, Xiap* and *A20*, thus providing the possibility that canonical NF-κB activation may account for induction and repression of target genes depending on the presence of coactivators, given cell type and induction mechanism [69]. There is also the possibility that NF-κB mediated changes in epigenetic gene regulation may affect Bdnf expression [71-73]. Moreover, IKK2 has been previously described to phosphorylate Bcl-xL, a mechanism associated with reduced expression of this gene in stressed, post-mitotic neurons [10]. A recent publication by Zhang et al. [45] addressed the role of IKK2/NF-κB signalling in the hypothalamus, which increases with aging and mediates suppression of hypothalamic-gonadotropin-releasing hormone (GnRH1) expression finally promoting systemic aging. They found that elevated IKK2 and NF-κB activity induces cJun/cfos and PKC levels, which are able to inhibit *Gnrh1* promoter activity. This mode of NF-κB-mediated inhibition of gene expression might also account for NF-κB-mediated Bdnf repression since Bdnf expression is known to be regulated by multiple promoters.

More importantly, the IKK2-CA transgene and Bdnf expression pattern do not coincide very well in the DG of IKK2^nCA^ mice (Figure 6D). Bdnf expression is located to the hilus region whereas IKK2-CA protein is detected in GCL neurons. In support of this, GABAergic interneurons present in the hilus are devoid of *Camk2a* expression thereby excluding Camk2a-driven transgene expression in these neurons [74]. This strongly argues for a scenario that IKK2-CA mediated NF-κB activation does not directly influence Bdnf expression. Rather, a so far unknown factor/s released by IKK2-CA positive neurons suppresses Bdnf production in a paracrine manner in the vicinity of the hilus, a process that necessarily does not depend on NF-κB-mediated gene regulation.

Notably, recent work by Han et al. reported that the *Camk2a-tTA* transgene, also used in the present study to drive IKK2-CA expression, itself exhibits a degenerating effect on the neurons which was not recognized by the scientific community for many years. Moreover, they observed that this degeneration was permanently rescued by administration of DOX during the first 6 weeks of life [75]. As the IKK2^nCA^ animals were treated with DOX up to the age of 4 weeks, most likely, we also avoid the tTA-induced degeneration. Consistent with that, the *Camk2a-tTA*-induced neurodegeneration gets obvious already at the age of 2 months, whereas a IKK2^nCA^ animals do not show atrophy up to the age of 3 months rather develop degeneration between 3 and 6 month age periods. Furthermore, IKK2^nCA^ animals were bred in pure NMRI background, an outbred model, which is different from the analysed hybrid strains sensitive for tTA-induced degeneration.

## Conclusion

In the present study we demonstrate that chronic activation of IKK2/NF-κB signalling in excitatory forebrain neurons does not induce a self-propagating inflammatory response including immune cell infiltration, as observed in other model systems. Instead, it interferes with spatial learning concomitant with decrease in Bdnf levels and neurodegeneration in the DG. Furthermore, we propose a novel mechanism of IKK/NF-κB dependent regulation of neuronal homeostasis and function, in particular a paracrine downregulation of Bdnf expression leading to impaired learning capabilities and dentate gyrus degeneration. Remarkably, our reverse remodelling results clearly show the high structural plasticity of the DG even in elderly animals. To what extent Bdnf depletion by enhanced neuronal IKK2/NF-κB activation is relevant to the pathogenesis of neurological disorders is an interesting question arising from this study, which with further elucidation can provide valuable insights to develop therapeutic strategies for neurodegenerative diseases.

## Materials and methods

### Transgenic mice

Mice were kept in a specific pathogen-free (SPF) animal facility at University of Ulm. Double transgenic mice (CaMK2a-tTA x luciferase-(tetO)_7_-CA-IKK2) were generated by directly crossing CaMK2a-tTA mice with single transgenic mice carrying a luciferase-(tetO)_7_-IKK2-CA transgene. The latter mice have a bidirectional promoter (tetO)_7_ which regulates the expression of luciferase reporter gene as well as IKK2-CA [9]. Both single transgenic mouse lines were bred on the NMRI background. In order to avoid any interference with brain development, inactivation of transgene expression was carried out by administration of DOX (0.1 g/l, MP Biomedicals) in 1% sucrose, in the drinking water to the dams during pregnancy, and to pups until 4 weeks of age. As control animals usually (tetO)7-IKK2-CA single transgenic littermates were used. Genotyping was made by PCR.

All animal experiments were performed in compliance with the Guide for the Care and Use of Laboratory Animals published by the US National Institutes of Health and the German Animal Protection Act and was approved by the Regierungspräsidium Tübingen, Germany that is the responsible government agency for animal rights.

### In vivo bioluminescence assay

Images for brain localized transgene expression were obtained with IVIS 200 System (Caliper) by performing IVIS as described by [76] and [77].

### Luciferase assay

For the detection of transgene, the tissue samples were snap frozen in liquid nitrogen, and the extracts were prepared by homogenizing the pulverized tissue in TNT extraction buffer. Luciferase reporter assay was performed as previously described [76].

### Immunoblotting

Native tissue protein extracts were prepared as described previously [18]. Equal amounts (50 μg) of total proteins were resolved on SDS-PAGE gels and transferred to nitrocellulose membranes by a standard western blot protocol. Membranes were then blocked with 5% non-fat dry milk in TBS buffer for 1 h at room temperature. Incubation with primary antibody (see below) was performed in blocking solution overnight at 4°C or for 2 h at room temperature. After washing with TBS, incubation with the HRP coupled secondary antibody was performed for 1 h at room temperature.

Membranes were exposed to ECL detection reagent from Invitrogen for the detection of signals. The “Intelligent Dark Box” (Fuji) was used to reveal the luminescence signals.

### Histology and immunostaining

For the histopathological analysis, animals were perfused with PBS and 4% PFA and decapitated. Brains were then fixed by immersion in 4% PFA (3-4 h at room temperature), dehydrated, embedded in paraffin, and cut to 7 μm thick coronal sections using the microtome Microm HM355S (Thermo Scientific, Waldorf, Germany). For making cryosections, the brains were frozen as described by [76] and the frozen brains were sectioned to 8 μm thick slices using cryotome Leica CM1900 (Leica Microsystems, Wetzlar, Germany). To identify the morphology of brain, nissl staining was performed with the paraffin sections. For immunofluorescent staining, after rehydration, heat mediated antigen retrieval was performed with sodium citrate (10 mM, pH 6, 0.05% Tween 20) or Tris-EDTA (10 mM Tris, 1 mM EDTA, pH 9, 0.05% Tween 20) and for full permeabilization sections were incubated with 0.5% Triton X-100 for 30 min. Sections were washed with PBS and blocked with 5% BSA with Fc Block antibody (BD Pharmingen, dilution 1:100) for 1h. Incubation with the primary antibodies (in 5% BSA) was performed overnight at 4°C, secondary antibodies were applied for 1h at room temperature with DAPI for nuclear counterstaining. For GFAP, cleaved caspase-3 and CD45 staining, the cryosections from natively frozen brains were fixed with cold methanol (-20°C). Blocking and staining was performed as described above. Fluorescence images were acquired with the Zeiss Axiovert 200 M microscope with filters for DAPI, FITC/Alexa Fluor 488, and TexasRed/Alexa Fluor 568/594 and the Zeiss Axiovision software. For every channel exposure times were adjusted separately and kept same for the complete session. Adjustment of contrast and brightness was performed distinctly for each channel, but equally in all compared pictures.

For immunohistochemistry, paraffin sections were treated with 3% hydrogen per oxide, heat mediated antigen retrieval was performed with citric acid buffer (pH 6.0), washed with TBS and blocked with 5% BSA for 1h at room temperature. Afterwards, slides were incubated with the primary antibodies against Iba1, RelA or Bdnf over night at room temperature. Biotinylated rabbit secondary antibody was applied for 30 min at room temperature, subsequently slides were treated with streptavidin HRP and the signals for Iba-1 were obtained using DAB, whereas by AEC reagent for RelA and Bdnf.

Fluoro-jade B staining was carried out with the cryosection of animals perfused with 4%PFA as described by [78].

Images were obtained by Leica CTR5500 microscope.

### Antibodies for immunostaining and immunoblotting

Goat anti-human IKK2 (sc-7329), rabbit anti-RelA (sc-372), rabbit anti-Bdnf (sc-546), rabbit anti-Erk2 (sc-154) and HRP-conjugated goat anti-rabbit or donkey anti-goat were obtained from Santa Cruz Biotechnology. Mouse anti-NeuN from Millipore (MAB 377), rabbit anti-GFAP from Abcam (Ab56777), rabbit anti-Cleaved-caspase3 from cellsignalling (9661), rat anti-CD45 from BD Pharmingen (BD550539) and rabbit anti-Iba1 was obtained from WAKO (019-19741).

Alexa Fluor labelled secondary antibodies were obtained from Invitrogen, DAPI was purchased from MERCK, and biotinylated anti-rabbit from VECTOR Laboratories U.S.A.

### TUNEL assay

TUNEL Assay was performed with paraffin sections using the Calbiochem TUNEL Assay kit, according to the manufacturer’s instructions.

### Kinase assay

Kinase assay was performed to measure basal IKK activity. The IKK complex was immunoprecipitated from striatal lysates with an antibody recognizing NEMO using protein A beads. The *in-vitro*-kinase assay was done as described in [9], taking recombinant GST-IκBα as substrate. Radiolabelled ATP was used, whose γ-Phosphate is transferred to the substrate GST-IκBα in the presence of the IKK complex proportional to its activity. Kinase activity was determined by detection of radiolabelled GST-IκBα after SDS-PAGE and western blot. For loading control, NEMO levels were detected in the precipitates by immunoblot.

### RNA extraction, cDNA synthesis and qPCR

RNA from hippocampus and cortex was isolated with the PeqGOLD Trifast (peQlab) kit from the frozen tissue pulverized with a morter and pestle under liquid nitrogen. cDNA was synthesized using Roche Transcriptor High fidelity cDNA synthesis kit with 0.8 μg of total RNA and oligo-dT-primers according to the manufacturer’s instructions. Quantitative Realtime-PCR assays were performed with the Lightcycler 480 Instrument (Roche Applied Science) with primers and hydrolysis probes designed by the Roche Universal Probe Library system. Hypoxanthine-guanine phosphoribosyltransferase gene (*Hprt)* was used as housekeeping gene.

Primer sequences and UPLs used for the quantitative real time PCR are as follows: *Hprt* (5′-GGA GCG GTA GCA CCT CCT-3′, 5′- CCT GGT TCA TCA TCG CTA ATC-3′, UPL no. 69), *Tnf* (5′- TGC CTA TGT CTC AGC CTC TTC-3′, 5′- GAG GCC ATT TGG GAA CTT CT-3′, UPL no. 49), *Ccl2* (MCP1) (5′- CAT CCA CGT GTT GGC TCA-3′, 5′- GAT CAT CTT GCT GGT GAA TGA GT-3′), *Cxcl10* (IP10) (5′- GCT GCC GTC ATT TTC TGC-3′, 5′ TCT CAC TGG CCC GTC ATC-3′ UPL no. 3), *Ptgs2* (cycloxygenase 2) (5′- GAT GCT CTT CCG AGC TGT G-3′, 5′- GGA TTG GAA CAG CAA GGA TTT - 3′, UPL no. 45), *Bdnf* (5′-AGT CTC CAG GAC AGC AAA GC-3′, 5′-TGC AAC CGA AGT ATG AAA TAA CC-3′, UPL no. 31), *Ngf-β* (5′-AAT TAG GCT CCC TGG AGG TG-3′, 5′-TGG ACT GCA CGA CCA CAG-3′, UPL no. 22), *Ntf3* (5′-CGA CGT CCC TGG AAA TAG TC-3′, 5′-TGG ACA TCA CCT TGT TCA CC-3′, UPL no. 29), *Igf2* (5′-CGC TTC AGT TTG TCT GTT CG-3′, 5′-GCA GCA CTC TTC CAC GAT G-3′, UPL no. 40), *Prkaca* (PKA catalytic α) (5′-GGC TCT CGG AGT CCT CAT C-3′, 5′-CAG AGC TGA AGT GGG ATG G-3′, UPL no. 46) *Gria1* (GluR1) (5′- AGG GAT CGA CAT CCA GAG AG-3′, 5′- TGC ACA TTT CCT GTC AAA CC-3′, UPL no. 62), *Gria2* (GluR2) (5′- CCA ATG GGA TAA GTT CGC ATA-3′, 5′- GCA CAG CTT GCA GTG TTG A-3′, UPL no. 110), *Gria3* (GluR3) (5′- AAG CCG TGT GAT ACG ATG AA-3′, 5′- TGC CAG GTT AAC AGC ATT TCT-3′, UPL no. 31), *Gria4* (GluR4) (5′- CTG CCA ACA GTT TTG CTG TG-3′, 5′- AAA TGG CAA ACA CCC CTC TA -3′, UPL no. 48), *Bcl2* (5′- GTA CCT GAA CCG GCA TCT G -3′, 5′-GGG GCC ATA TAG TTC CAC AA-3′, UPL no. 75), *Bcl2l1* (Bcl-xL) (5′- TGA CCA CCT AGA GCC TTG GA - 3′, 5′- TGT TCC CGT AGA GAT CCA CAA - 3′, UPL no. 2), *Il6* (5′- CT ACC AAA CTG GAT ATA ATC AGG A - 3′, 5′- CCA GGT AGC TAT GGT ACT CCA GAA-3′, UPL no. 6),), *Ccl5* (RANTES) (5′- TGC AGA GGA CTC TGA GAC AGC - 3′, 5′- GAG TGG TGT CCG AGC CATA - 3′, UPL no. 110), *Tdo2* (5′- AAT CAG AGC AGG AGC AGA CG - 3′, 5′- TTG GCT CTA AAC CAG GTG TTC -3′, UPL no. 22), *Mrg1b* (5′- AGA CAA GGA CGC AAT CTA TGG - 3′, 5′- GCT CGC ACT TCT CAA AAA CC -3′, UPL no. 6) and *Lcn2* (5′- CCA TCT ATG AGC TAC AAG AGA ACA AT - 3′, 5′- TCT GAT CCA GTA GCG ACA GC -3′, UPL no. 58).

### BDNF quantification

BDNF positive area was measured using ImageJ64 software. BDNF negative area was cut from the photomicrographs, subsequently, quantification was made at a particular threshold level by measuring BDNF positive area in the specific (red) channel.

### Quantification of astrogliosis

GFAP positive area was measured using ImageJ64 software. The background was subtracted after importing the images in ImageJ64. Similar threshold level was set for every image, on the dark background (in the particular channel; texas red) and the positive signals were quantified.

### Quantification of microglia and Ki67 positive cells

Iba1-positive microglia were counted in two fields of cortex, CA1 region and dentate gyrus per mouse. Ki67-positive cells were counted in two fields of dentate gyrus for every animal in each age group. Images were obtained using Leica CTR5500 microscope. Cell count was performed manually using ImageJ64 software.

### Quantification of nuclear cells in the dentate gyrus and CA1 region

Cresyl violet stained images were obtained from control and transgenic mouse brain sections. Particular areas were defined in the CA1 region and in the upper and lower blades of the dentate gyrus for quantification of cell number. Nine coronal planes were selected from rostral to caudal part of brain (bregma: ~ 1.46, 1.70, 1.82, 2.06, 2.18, 2.30, 2.54, 2.80, and 3.08 mm according to ref. [79] to ensure similar topography and avoid errors due to the differences in orientation of planes. Cells were counted in the specified areas of matched planes using ImageJ64 software. Percentage cell loss was determined with respect to the cell number in the control animals. Primary cortex was measured (bregma: ~ 4.98 mm) with ImageJ64. Images for olfactory bulbi were taken at bregma: ~ 4.28 mm.

### Bdnf ELISA

ELISA for Bdnf was performed with Promega (Madison, WI, USA) ELISA kit. Protein extracts from cortex and hippocampus were made in the lysis buffer as described by Promega (1% Nonidet P-40, 20 mM Tris, pH 8.0, 137 mM NaCl, 10% glycerol, 1 mM phenyl-methylsulfonyl fluoride, protease inhibitor Complete mini (Roche), 0.5 mM sodium vanadate). The procedure was made according to the manufacturer’s instructions.

### Morris water maze task

The experimental subjects were 6 and 9 month old male mice (Co group, n = 13 IKK2^nCA^, (n = 13), which were housed individually in a 12 h light/dark schedule for one week before the Morris water maze (MWM) task was performed to get familiar with the place. Mice were habituated to handling by the experimenter at least for three days before the experiment. The experiment was performed as described by Vorhees and Williams [34] with some modifications; the animals were exposed to this spatial learning paradigm for eight consecutive days with four trials per mouse each day with an inter trial interval of 15 min. Visual cues were provided outside the pool on the walls of the room to help in navigation. Position of platform was kept same throughout the training session, however, each day the start locations for the trials were random. For all days, the experiment was performed at the same time of the day with the same environmental conditions. The room was sound proof and the experimenter was blind about the genotypes of mice. Before the MWM task, a visible platform test was performed to exclude motor and visual acuity impairment. The platform was marked with a flag, and tracking length and latency to reach the platform were recorded.

All data including track length, position of the animals and latency to find the hidden platform were recorded using an automated video tracking and analysis system, Viewer II software, (Biobserve, Bonn, Germany).

### Statisticical analysis

Statistical analysis was performed with the Prism-software (Graphpad). All data are shown as mean ± SEM. Statistical significances were determined by using unpaired Student’s *t* test or as stated in the figure legends. (* *p* < 0.05; ** *p* < 0.01; *** *p* < 0.001).

## Abbreviations

IKK: IκB kinase; NF-κB: Nuclear factor-kappa B; DAPI: 4*'*, 6-diamidino-2-phenylindole; ELISA: Enzyme-linked immunosorbent assay.

## Competing interests

All authors of the manuscript declare that none of the authors have any financial interest related to this work.

## Authors’ contribution

AM carried out in vivo imaging, luciferase assays, western blot analysis, immunofluorescent staining, immunohistochemistry, Nissl staining, performed cell counting, qRT-PCR, ELISA and MWM, and drafted the manuscript. ML conducted experiments on microgliosis, helped to conceive the study and drafted the manuscript. TW contributed to the design and conception of experiments, and drafted the manuscript. BB carried out breeding of animals, conceived the study, participated in the design and coordination, and drafted the manuscript. All authors read and approved the final manuscript.

## Supplementary Material

Additional file 1 **IKK2-CA expression results in nuclear RelA localization in different forebrain-regions.** Immunohistochemistry of RelA depicts enhanced nuclear localisation of RelA in the DG, CA1-region, cortex and striatum of transgenic mice compared to controls (Age = 9M). Co = wild type and single transgenic littermate controls, IKK2^nCA^ = transgenic mice. Scale bar: 50 μm. (n = 3). Click here for file

Additional file 2 **Tnf-α immunoreactivity is increased in the DG of IKK2**^**nCA **^**mice.** (A) qRT-PCR analysis indicates that *Ccl5* and *Il6* are not deregulated in the hippocampal mRNA of IKK2^nCA^ mice at any timepoint. ns = non-significant. *P*-values are derived from two-tailed-unpaired student’s *t* test. (B) Representative images of Tnf-α (green) immunofluorescence stainings showing stronger immunoreactivity in the hilar neurons (NeuN) of the transgenic dentate gyrus (DG) as compared to the control littermates at the age of 3 months. (Coexpression of Tnf-α and NeuN is shown in yellow). Sections are costained with DAPI (blue) for visualising the nuclei. Inserts depict the magnification of the marked areas (n = 4). Scale bar: 50 μm. (C) qRT-PCR analysis of *Tdo2* (DG marker gene) and *Mrg2b* (CA1 marker gene) assure correct subregion isolation. The levels of *Tnf* are specifically high in the DG of IKK2^nCA^ mice, *Cxcl10* shows a tendency of upregulation in both DG and CA*,* whereas *Ccl2* and *Il6* are not deregulated in either region of IKK2^nCA^ mice (n = 6). *P*-values are derived from two-tailed-unpaired student’s *t* test. Co = wild type and single transgenic littermate controls, IKK2^nCA^ = transgenic mice with neuron-specific expression of constitutively active IKK2. All data are shown as mean ± SEM. ** *p* < 0.05, ** *p* < 0.01, *** *p* < 0.001. Click here for file

Additional file 3 **Analysis of infiltration of CD45-positive cells.** (A-C) Kryosections stained for CD45 do not reveal obvious immune cell infiltration in the IKK2^nCA^ model. Photomicrographs of 3, 6, 9M old control and IKK2^nCA^ mice indicate CD45^+^ cells in the (green). DAPI (blue) costaining is used for visualising the nuclei. Inserts at the right bottom depict the magnification of the marked areas. Scale bar: 50 μm, (n = 3-4). (D) mRNA analysis of *Lcn2* shows no upregulation in the DG or CA1-region of IKK2^nCA^ mice as compared to the control littermates. Co = wild type and single transgenic littermate controls, IKK2^nCA^ = transgenic mice with neuron-specific expression of constitutively active IKK2 (n = 3-4). Click here for file

Additional file 4 **Subregional analysis of learning-associated genes.** qRT-PCR analyses indicate DG-specific downregulation of *Bdnf* and *Gria3* in IKK2^nCA^ mice. However, *Gria1* levels are reduced in both DG and CA1-region, whereas *Prkaca* is not deregulated in the IKK2^nCA^ mice as compared to the littermate controls (age = 9M). Co = wild type and single transgenic littermate controls, IKK2^nCA^ = transgenic mice with neuron-specific expression of constitutively active IKK2 (n = 6). Click here for file

Additional file 5 **IKK2**^**nCA **^**mice do not show apoptosis in the CNS.** (A-C) Analysis of apoptosis by cleaved caspase-3 staining. Photomicrographs from the DG, hippocampus CA1 and cortex of 3, 6, 9M old control and IKK2^nCA^ mice indicate no caspase-3 (red) positive cells. Sections are stained with DAPI (blue) for visualising the nuclei (n = 4). (D) TUNEL assay was performed with paraffin sections from the hippocampus of age-matched control and transgenic mice at the age of 3, 6, and 9M. Similar number of TUNEL-positive cells was observed between both genotypes. Inserts at the right bottom demonstrate the magnification of the marked areas (n = 4). Co = wild type and single transgenic littermate controls, IKK2^nCA^ = transgenic mice with neuron-specific expression of constitutively active IKK2. Scale bar: 50 μm. Click here for file

Additional file 6 **Fluoro-jade B staining indicates degeneration of neurons in the DG of IKK2**^**nCA **^**mice.** (A-C) Fluoro-jade (green) positive cells and neurites are indicated. Sections are stained with DAPI (blue) for visualising the nuclei. Arrow heads point towards the degenerating neurites, whereas the arrows point towards the degenerating neurons. Inserts depict the magnification of the marked areas. (D) Fluoro-jade (green) positive cells and neurites are counted in the DG, CA1, and cortex of transgenic mice and control littermates. Dentate gyrus of IKK2^nCA^ mice shows a high number of degenerating cells and their processes as marked by FJ-B staining in all age groups. (E) Olfactory bulbs of control and IKK2^nCA^ mice are deficient of Fluoro-jade positive cells. (F) qRT-PCR analysis for *Bdnf* and *Tnf* expression reveals no changes in the olfactory bulbs. Co = wild type and single transgenic littermate controls, IKK2^nCA^ = transgenic mice with neuron-specific expression of constitutively active IKK2. Scale bar: 100 μm. (n = 4). * *p* < 0.05, ** *p* < 0.01, ns: non-significant. *P*-values are derived from two-tailed-unpaired student’s *t* test. Click here for file

Additional file 7 **IKK2-CA expression in cortex and olfactory bulbi does not result in structural degeneration.** (A) Photomicrographs from the nissl-stained cortex sections of 3, 6, 9M old control and IKK2^nCA^ mice. Black bars depict area for cortical thickness measurement. (B) Quantification of cortical thickness measurement by ImageJ64 shows no changes among the control and IKK2^nCA^ mice in the analysed age groups (n = 6-7 per age group). (C) Images of olfactory bulbi of 3, 6, 9M old control and IKK2^nCA^ mice. Glomerular layer (GL), rostral migratory stream (RMS), External plexiform layer (EPL), mitral cell layer (MCL), granule cell layer (GCL). (D) Thickness of GCL of olfactory bulbi was measured using ImageJ64. IKK2^nCA^ mice do not exhibit significant alterations in the GCL as compared to the control mice (n = 4-7). (E) Subregional analysis of *Bcl2* and *Bcl2l1* expression by qRT-PCR. Reduced levels in the DG and CA-region of IKK2^nCA^ = were detected for *Bcl2l1* compared to the littermate controls at the age of 9M*. Bcl2* expression shows a tendency of reduction (n = 6). Co = wild type and single transgenic littermate controls, IKK2^nCA^ = transgenic mice with neuron-specific expression of constitutively active IKK2. * *p* < 0.05, ** *p* < 0.01. Scale bar: A = 100 μm, C = 50 μm. Click here for file

Additional file 8 **Transgene inactivation increases neurogenesis in IKK2**^**nCA **^**mice.** (A) Ki67 immunostaining of paraffin sections depicts Ki67-positive cells in the GCL of IKK2^nCA^ mice after 3 months of DOX treatment. Inserts demonstrate magnified images of the marked areas (n = 4). (B) Quantification of Ki67-positive cells in the GCL reveals a significant higher number in the DOX-treated IKK2^nCA^ mice as compared to untreated age-matched IKK2^nCA^ mice. (Co = wild type and single transgenic littermate controls, IKK2^nCA^ = transgenic mice with neuron-specific expression of constitutively active IKK2. Scale bar: 50 μm. * *p* < 0.05, ** *p* < 0.01, ns: non-significant. *P*-values are derived from two-tailed-unpaired student’s *t* test. Click here for file
