# Supplementary figures and images for: Sustained, neuron-specific IKK/NF-κB activation generates a selective neuroinflammatory response promoting local neurodegeneration with aging
Source: Mol Neurodegener. 2013 Oct 12;8:40. doi: 10.1186/1750-1326-8-40 (PMC3827934; doi:10.1186/1750-1326-8-40)

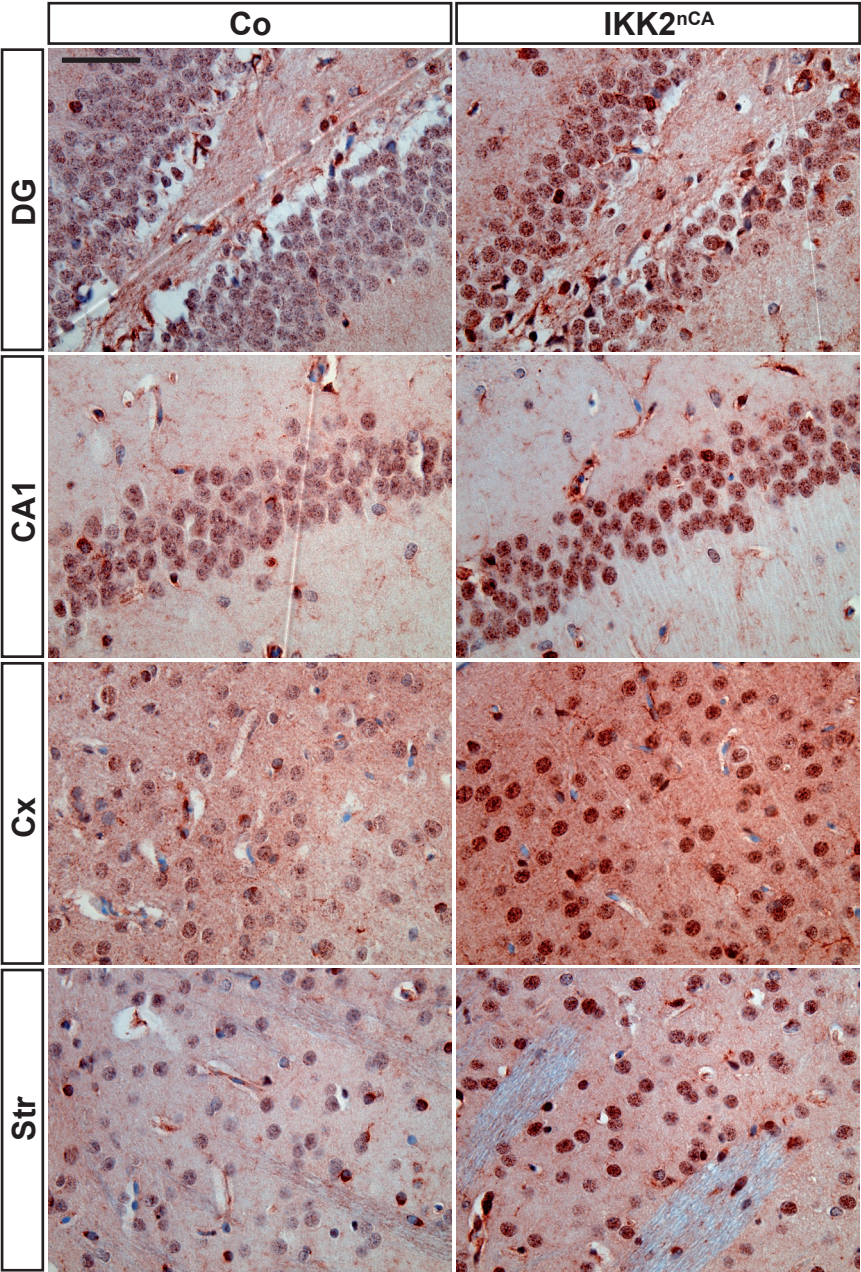

Supplement: Additional file 1 — IKK2-CA expression results in nuclear RelA localization in different forebrain-regions. Immunohistochemistry of RelA depicts enhanced nuclear localisation of RelA in the DG, CA1-region, cortex and striatum of transgenic mice compared to controls (Age = 9M). Co = wild type and single transgenic littermate controls, IKK2nCA = transgenic mice. Scale bar: 50 μm. (n = 3). [file 1750-1326-8-40-S1.pdf]

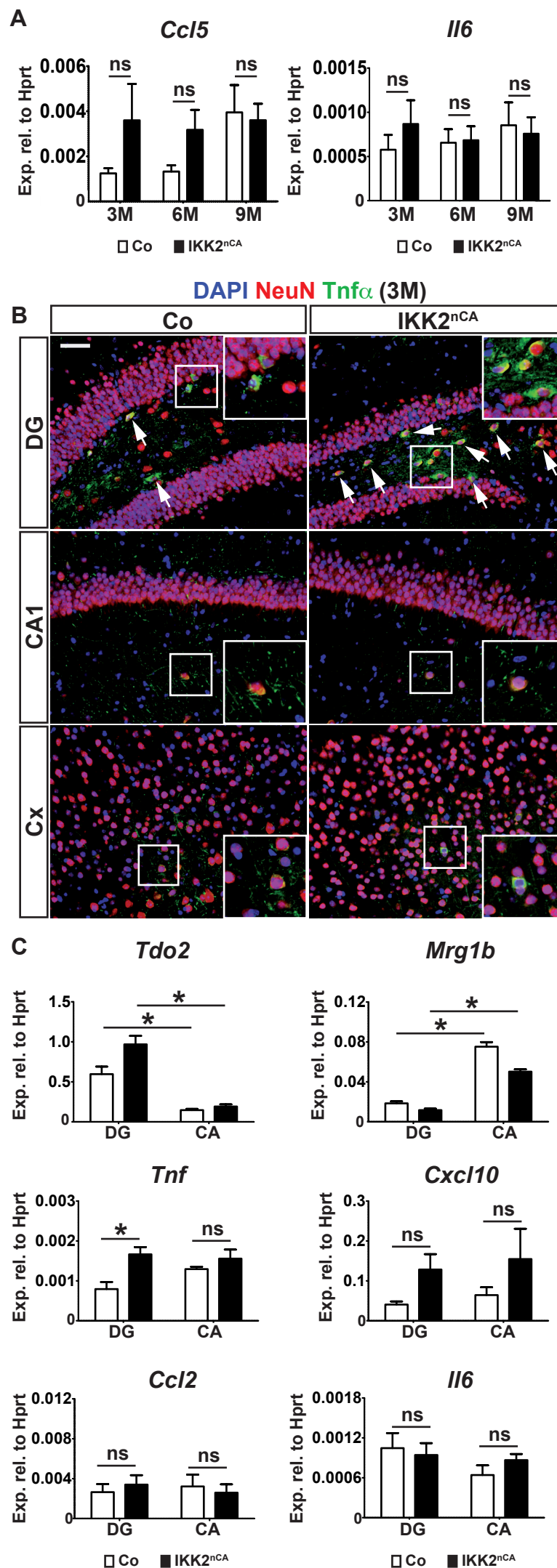

Supplement: Additional file 2 — Tnf-α immunoreactivity is increased in the DG of IKK2nCA mice. (A) qRT-PCR analysis indicates that Ccl5 and Il6 are not deregulated in the hippocampal mRNA of IKK2nCA mice at any timepoint. ns = non-significant. P-values are derived from two-tailed-unpaired student’s t test. (B) Representative images of Tnf-α (green) immunofluorescence stainings showing stronger immunoreactivity in the hilar neurons (NeuN) of the transgenic dentate gyrus (DG) as compared to the control littermates at the age of 3 months. (Coexpression of Tnf-α and NeuN is shown in yellow). Sections are costained with DAPI (blue) for visualising the nuclei. Inserts depict the magnification of the marked areas (n = 4). Scale bar: 50 μm. (C) qRT-PCR analysis of Tdo2 (DG marker gene) and Mrg2b (CA1 marker gene) assure correct subregion isolation. The levels of Tnf are specifically high in the DG of IKK2nCA mice, Cxcl10 shows a tendency of upregulation in both DG and CA, whereas Ccl2 and Il6 are not deregulated in either region of IKK2nCA mice (n = 6). P-values are derived from two-tailed-unpaired student’s t test. Co = wild type and single transgenic littermate controls, IKK2nCA = transgenic mice with neuron-specific expression of constitutively active IKK2. All data are shown as mean ± SEM. ** p < 0.05, ** p < 0.01, *** p < 0.001. [file 1750-1326-8-40-S2.pdf]

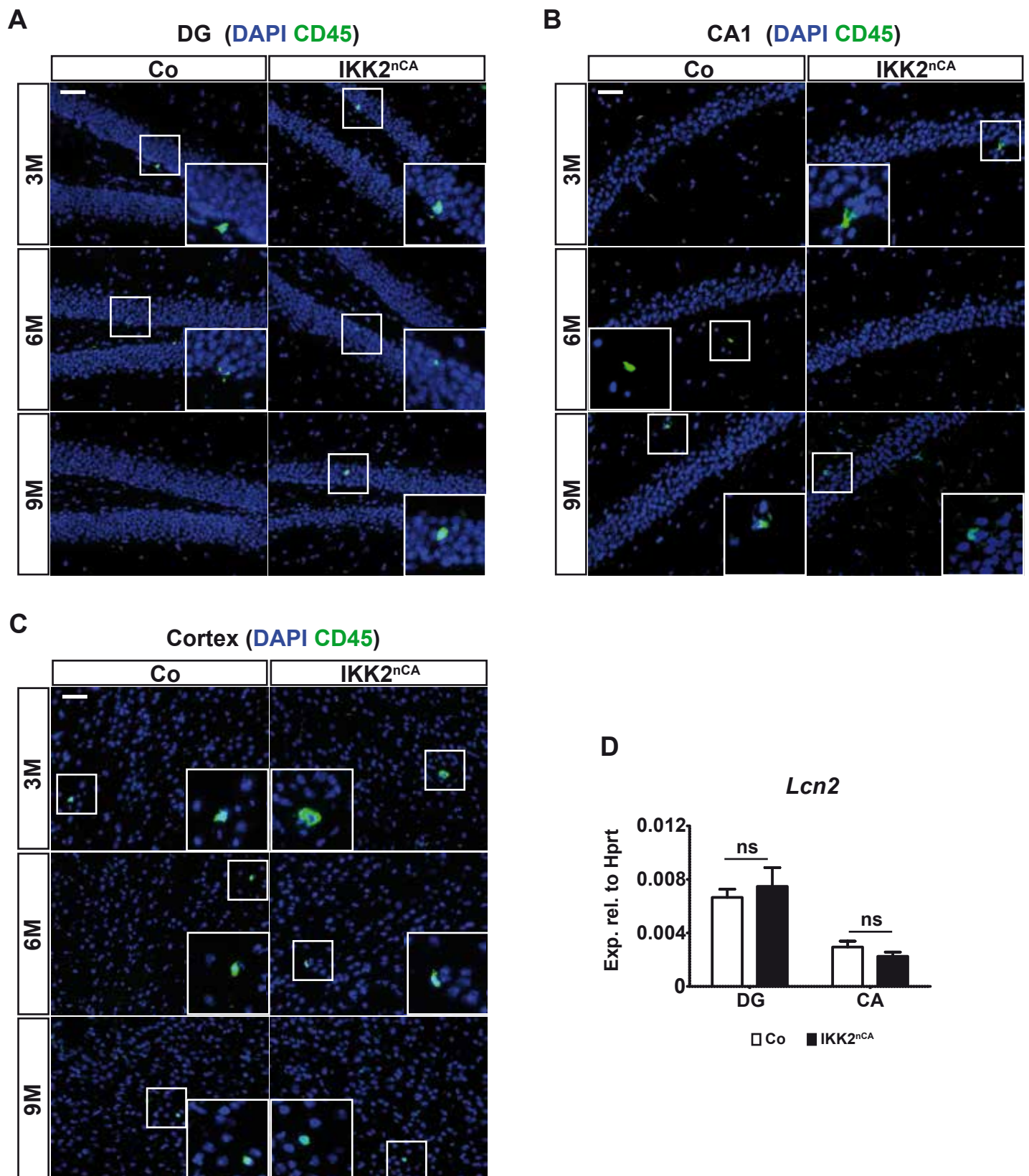

Supplement: Additional file 3 — Analysis of infiltration of CD45-positive cells. (A-C) Kryosections stained for CD45 do not reveal obvious immune cell infiltration in the IKK2nCA model. Photomicrographs of 3, 6, 9M old control and IKK2nCA mice indicate CD45+ cells in the (green). DAPI (blue) costaining is used for visualising the nuclei. Inserts at the right bottom depict the magnification of the marked areas. Scale bar: 50 μm, (n = 3-4). (D) mRNA analysis of Lcn2 shows no upregulation in the DG or CA1-region of IKK2nCA mice as compared to the control littermates. Co = wild type and single transgenic littermate controls, IKK2nCA = transgenic mice with neuron-specific expression of constitutively active IKK2 (n = 3-4). [file 1750-1326-8-40-S3.pdf]

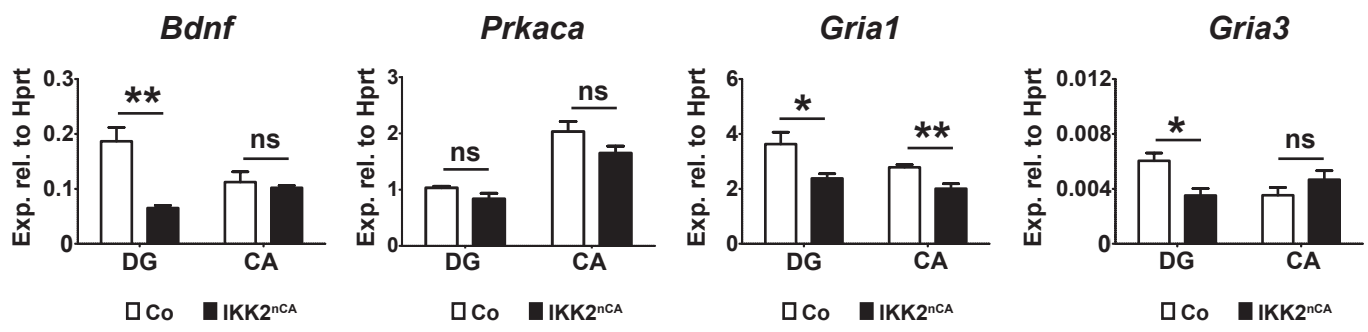

Supplement: Additional file 4 — Subregional analysis of learning-associated genes. qRT-PCR analyses indicate DG-specific downregulation of Bdnf and Gria3 in IKK2nCA mice. However, Gria1 levels are reduced in both DG and CA1-region, whereas Prkaca is not deregulated in the IKK2nCA mice as compared to the littermate controls (age = 9M). Co = wild type and single transgenic littermate controls, IKK2nCA = transgenic mice with neuron-specific expression of constitutively active IKK2 (n = 6). [file 1750-1326-8-40-S4.pdf]

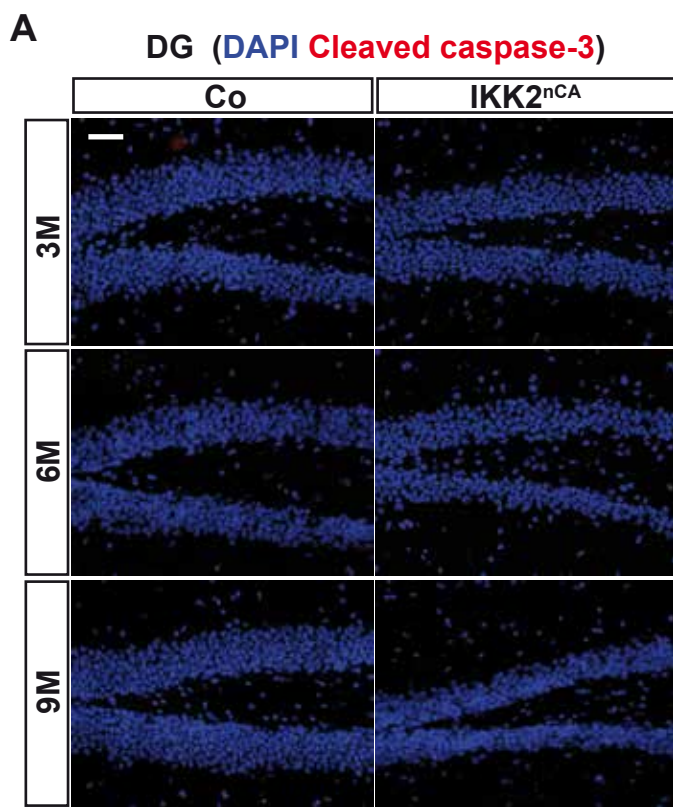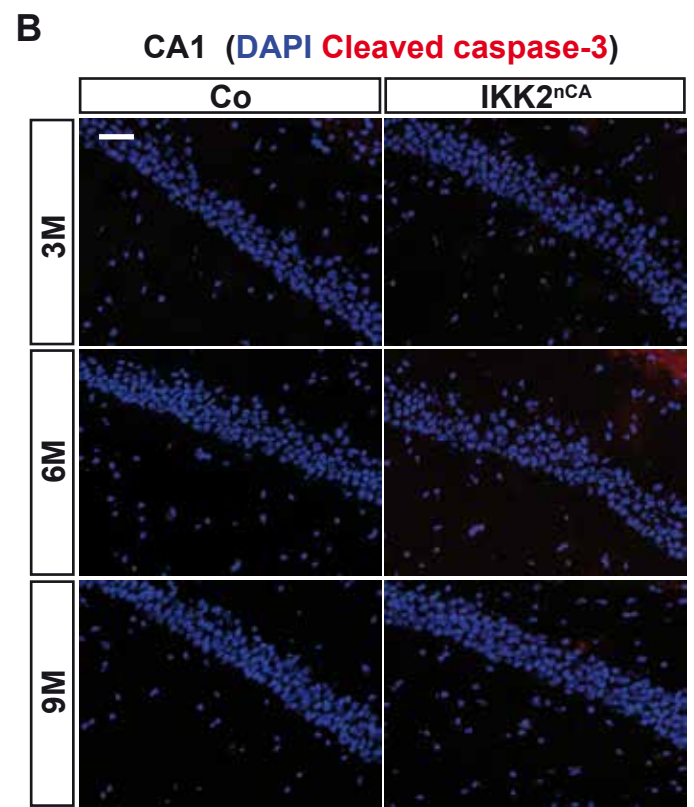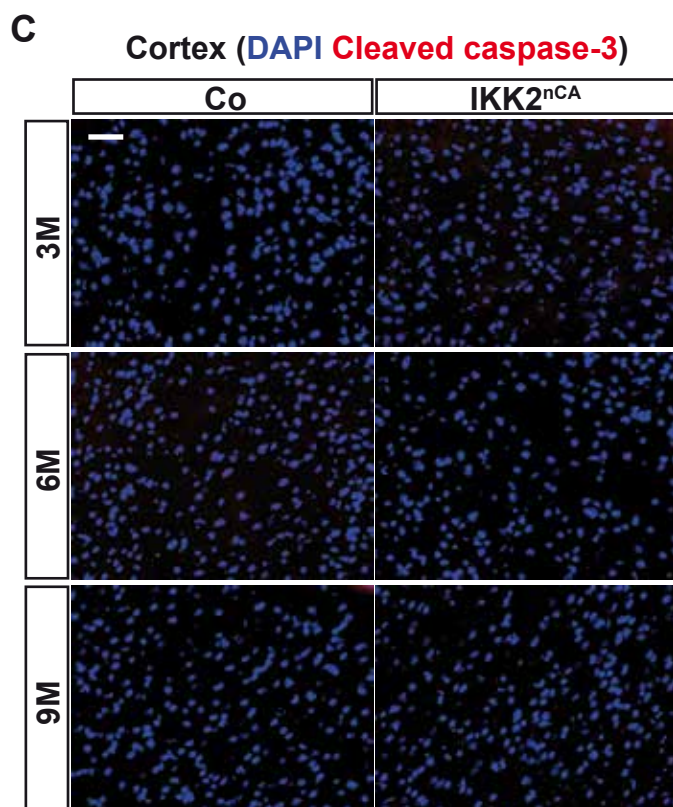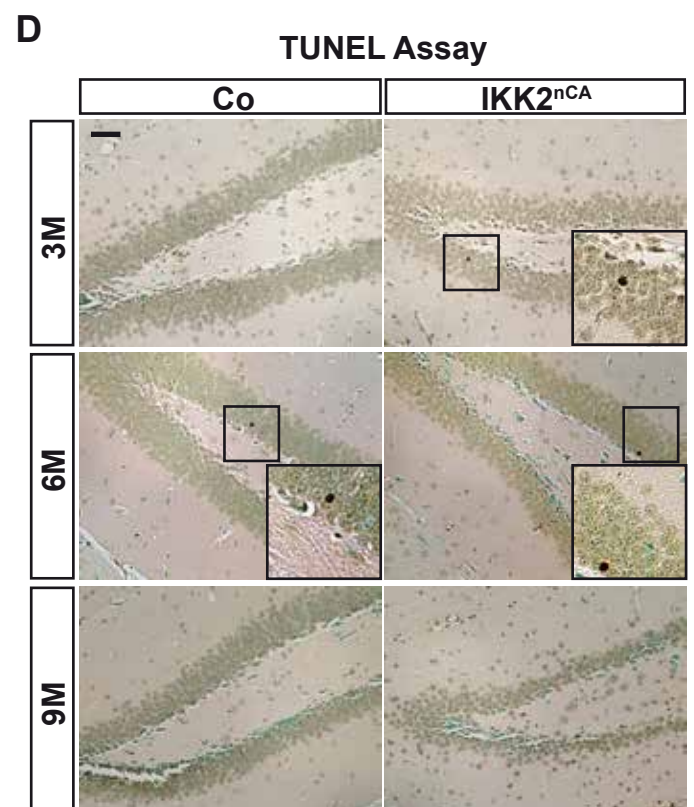

Supplement: Additional file 5 — IKK2nCA mice do not show apoptosis in the CNS. (A-C) Analysis of apoptosis by cleaved caspase-3 staining. Photomicrographs from the DG, hippocampus CA1 and cortex of 3, 6, 9M old control and IKK2nCA mice indicate no caspase-3 (red) positive cells. Sections are stained with DAPI (blue) for visualising the nuclei (n = 4). (D) TUNEL assay was performed with paraffin sections from the hippocampus of age-matched control and transgenic mice at the age of 3, 6, and 9M. Similar number of TUNEL-positive cells was observed between both genotypes. Inserts at the right bottom demonstrate the magnification of the marked areas (n = 4). Co = wild type and single transgenic littermate controls, IKK2nCA = transgenic mice with neuron-specific expression of constitutively active IKK2. Scale bar: 50 μm. [file 1750-1326-8-40-S5.pdf]

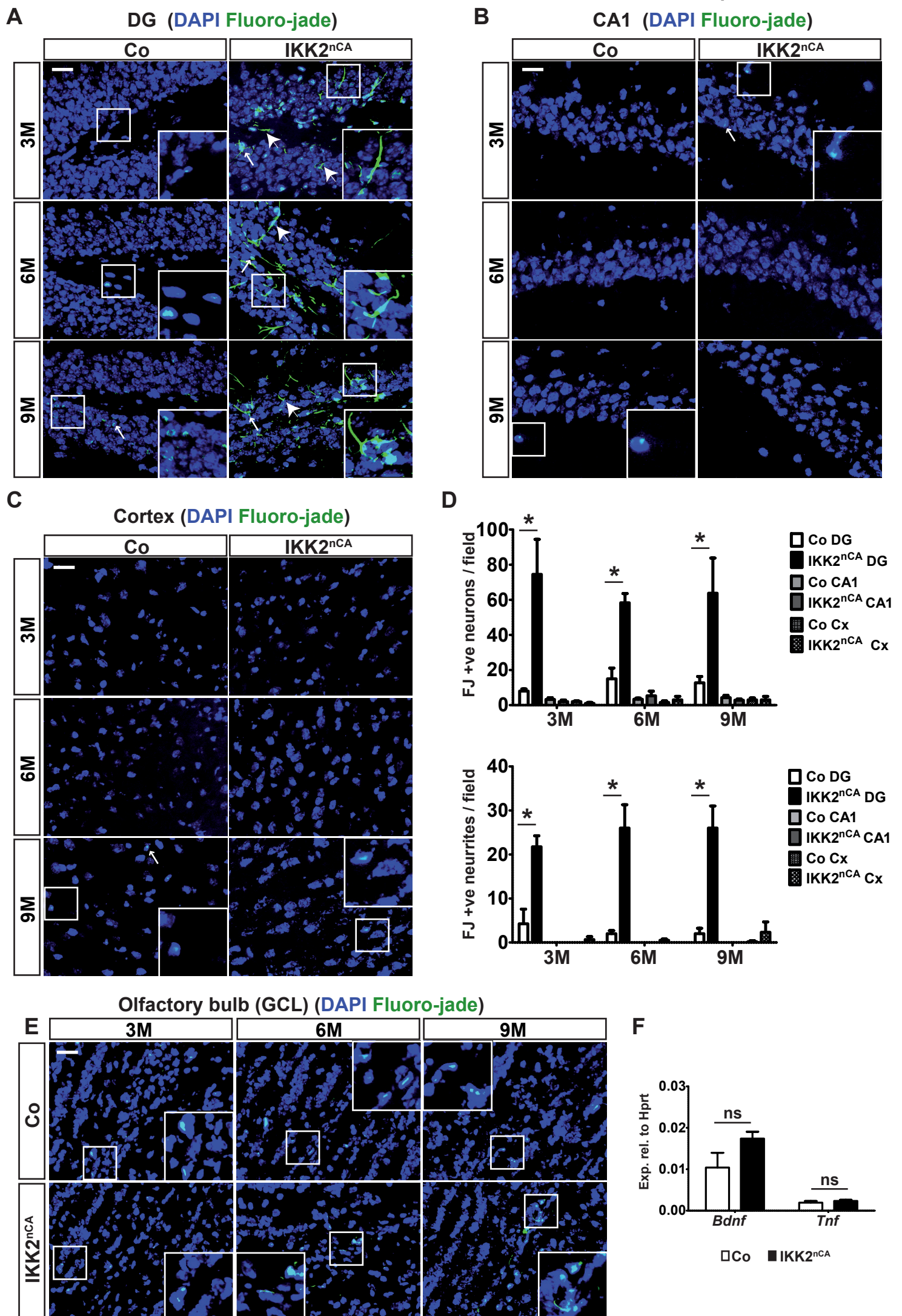

Supplement: Additional file 6 — Fluoro-jade B staining indicates degeneration of neurons in the DG of IKK2nCA mice. (A-C) Fluoro-jade (green) positive cells and neurites are indicated. Sections are stained with DAPI (blue) for visualising the nuclei. Arrow heads point towards the degenerating neurites, whereas the arrows point towards the degenerating neurons. Inserts depict the magnification of the marked areas. (D) Fluoro-jade (green) positive cells and neurites are counted in the DG, CA1, and cortex of transgenic mice and control littermates. Dentate gyrus of IKK2nCA mice shows a high number of degenerating cells and their processes as marked by FJ-B staining in all age groups. (E) Olfactory bulbs of control and IKK2nCA mice are deficient of Fluoro-jade positive cells. (F) qRT-PCR analysis for Bdnf and Tnf expression reveals no changes in the olfactory bulbs. Co = wild type and single transgenic littermate controls, IKK2nCA = transgenic mice with neuron-specific expression of constitutively active IKK2. Scale bar: 100 μm. (n = 4). * p < 0.05, ** p < 0.01, ns: non-significant. P-values are derived from two-tailed-unpaired student’s t test. [file 1750-1326-8-40-S6.pdf]

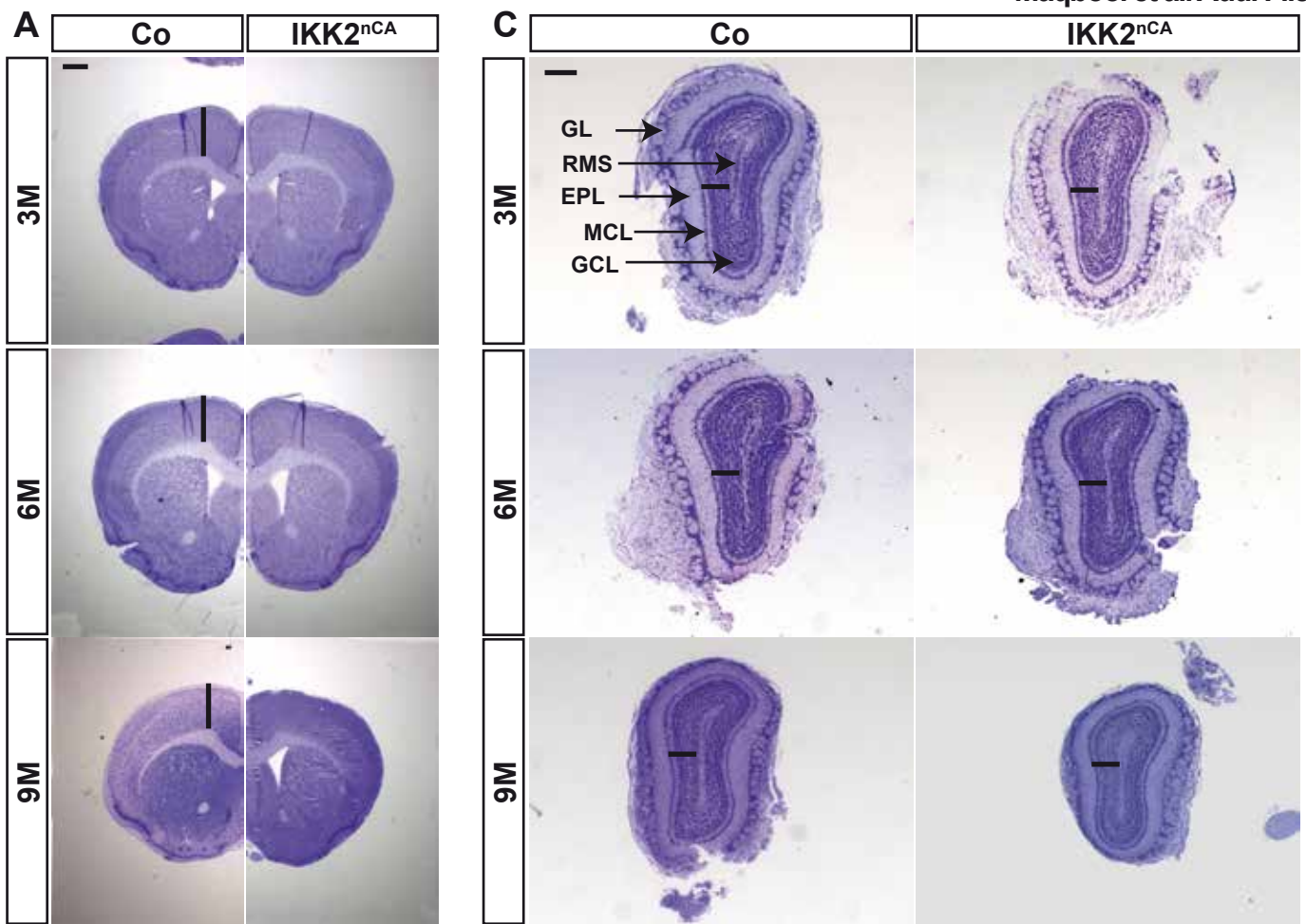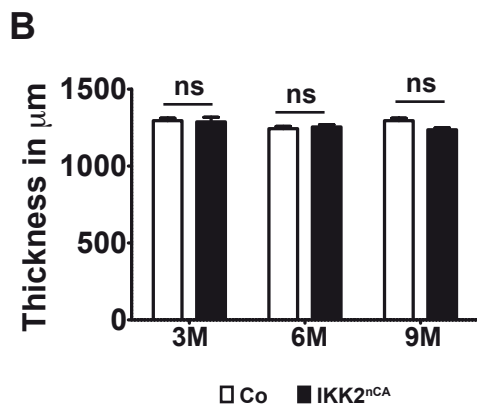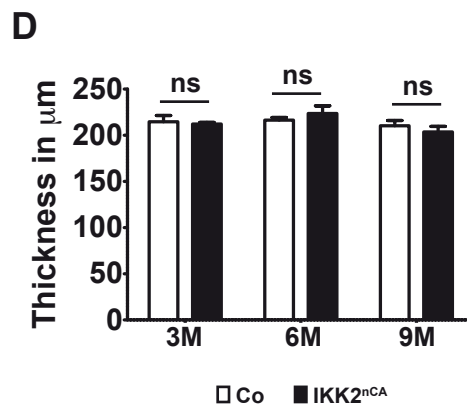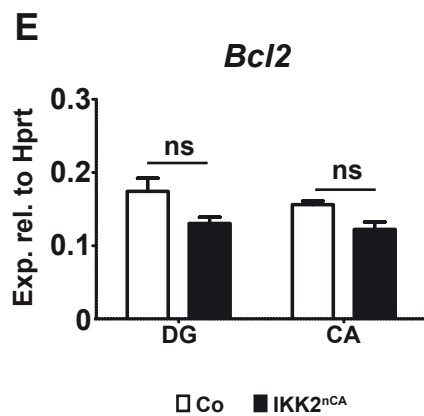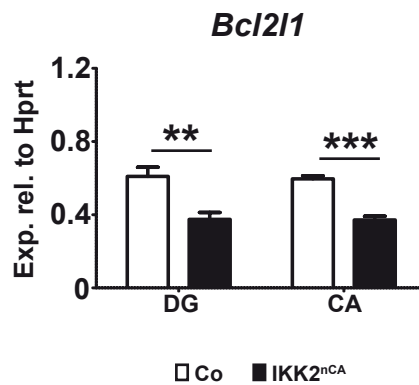

Supplement: Additional file 7 — IKK2-CA expression in cortex and olfactory bulbi does not result in structural degeneration. (A) Photomicrographs from the nissl-stained cortex sections of 3, 6, 9M old control and IKK2nCA mice. Black bars depict area for cortical thickness measurement. (B) Quantification of cortical thickness measurement by ImageJ64 shows no changes among the control and IKK2nCA mice in the analysed age groups (n = 6-7 per age group). (C) Images of olfactory bulbi of 3, 6, 9M old control and IKK2nCA mice. Glomerular layer (GL), rostral migratory stream (RMS), External plexiform layer (EPL), mitral cell layer (MCL), granule cell layer (GCL). (D) Thickness of GCL of olfactory bulbi was measured using ImageJ64. IKK2nCA mice do not exhibit significant alterations in the GCL as compared to the control mice (n = 4-7). (E) Subregional analysis of Bcl2 and Bcl2l1 expression by qRT-PCR. Reduced levels in the DG and CA-region of IKK2nCA = were detected for Bcl2l1 compared to the littermate controls at the age of 9M. Bcl2 expression shows a tendency of reduction (n = 6). Co = wild type and single transgenic littermate controls, IKK2nCA = transgenic mice with neuron-specific expression of constitutively active IKK2. * p < 0.05, ** p < 0.01. Scale bar: A = 100 μm, C = 50 μm. [file 1750-1326-8-40-S7.pdf]

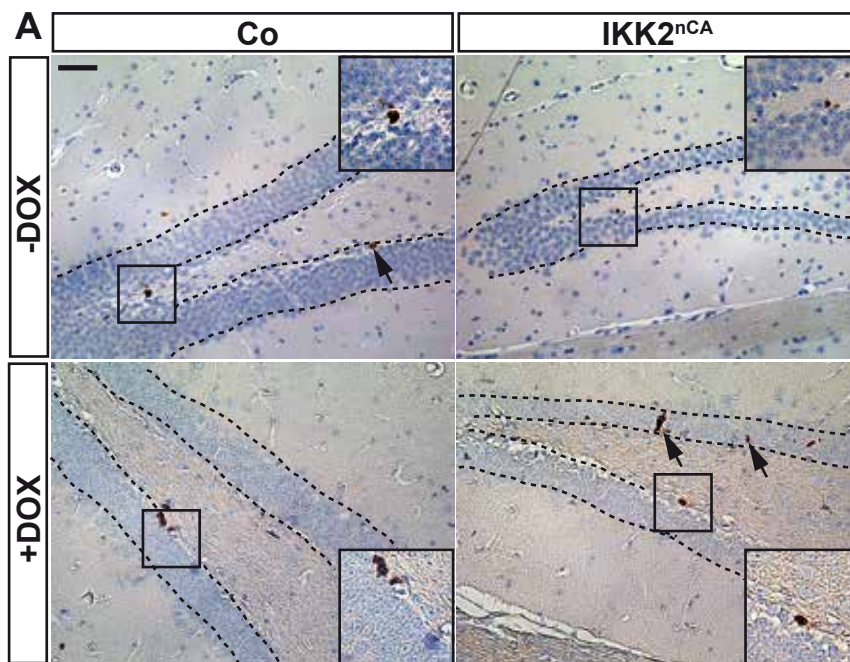

**B**

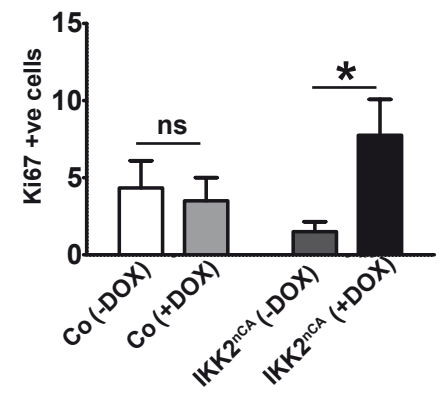

Supplement: Additional file 8 — Transgene inactivation increases neurogenesis in IKK2nCA mice. (A) Ki67 immunostaining of paraffin sections depicts Ki67-positive cells in the GCL of IKK2nCA mice after 3 months of DOX treatment. Inserts demonstrate magnified images of the marked areas (n = 4). (B) Quantification of Ki67-positive cells in the GCL reveals a significant higher number in the DOX-treated IKK2nCA mice as compared to untreated age-matched IKK2nCA mice. (Co = wild type and single transgenic littermate controls, IKK2nCA = transgenic mice with neuron-specific expression of constitutively active IKK2. Scale bar: 50 μm. * p < 0.05, ** p < 0.01, ns: non-significant. P-values are derived from two-tailed-unpaired student’s t test. [file 1750-1326-8-40-S8.pdf]
